# The Effector *SIX8* Contributes to Virulence of *Fusarium oxysporum* f. sp. *lactucae* Race 4 on Lettuce

**DOI:** 10.1111/mpp.70296

**Published:** 2026-06-09

**Authors:** Andrew D. Legg, Babette V. Vlieger, Petra Houterman, Martijn Rep, John P. Clarkson

**Affiliations:** ^1^ School of Life Sciences University of Warwick Warwick UK; ^2^ Molecular Plant Pathology University of Amsterdam Amsterdam the Netherlands

**Keywords:** CRISPR mutagenesis, effector, *Fusarium oxyporum*, lettuce, plant pathogen, secreted in xylem genes, *SIX8*

## Abstract

Fusarium wilt disease of lettuce caused by the soilborne fungus *Fusarium oxysporum* f. sp. *lactucae* (Fola) causes substantial crop losses worldwide. Four races of Fola have been identified with race 1 (Fola1) and the more recently emerged race 4 (Fola4) predominant in Europe. Recent analysis of Fola1 and Fola4 genomes alongside PCR‐based isolate profiling determined that while both Fola1 and Fola4 isolates harbour homologues of the putative effector genes *Secreted in Xylem (SIX) 9* and *SIX14*, Fola4 has additionally gained *SIX8*, which is divergently transcribed with *Pair with Six Eight 1* (*PSE1*) or *Pair with Six Eight 1*‐ *Like* (*PSL1*) genes. Transcriptomic analyses at a single timepoint also demonstrated high expression levels for *SIX8*, *SIX9*, *SIX14* and *PSL1* during Fola4 lettuce infection. In this study we demonstrate that *SIX8*, *SIX9* and *SIX14* expression levels increase up to 96 h following Fola4 lettuce infection, hence confirming their potential importance in wilt disease. The role of *SIX8* in virulence on lettuce was then investigated through phenotyping of knockout mutants generated by CRISPR‐Cas9 gene editing. Δ*SIX8* mutants resulted in reduced disease in both in vitro lettuce seedling and pot‐based lettuce plant glasshouse bioassays compared to wild‐type Fola4. Complementation of *SIX8* back into one of the knockout mutants using *Agrobacterium*‐mediated transformation restored full virulence on lettuce. We conclude that *SIX8* plays a role in Fola4 virulence and discuss the hypothesis that *SIX8* may allow Fola4 to overcome some sources of Fola1 resistance and hence infect a different range of lettuce cultivars.

## Graphical abstract

Secreted in Xylem (*SIX*) genes were expressed during infection of lettuce by *Fusarium oxysporum* f. sp. *lactucae* race 4 (Fola4). CRISPR‐Cas9 knockout of the *SIX8* gene in Fola4 reduced virulence in lettuce which was restored following *Agrobacterium* mediated complementation. *SIX8* therefore contributes to virulence of Fola4 on lettuce.

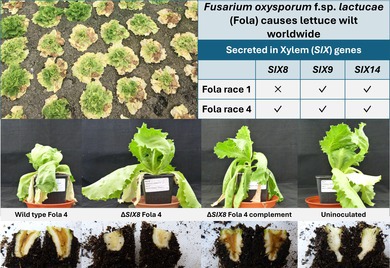

## 1 Introduction

*Fusarium oxysporum* is a globally distributed fungal species complex that includes pathogenic isolates adapted to infect different plant hosts. These can cause substantial losses on important crop plants with disease symptoms including crown rots, root rots and vascular wilts. Pathogenic *F. oxysporum* isolates are grouped into host‐specific *formae speciales* (ff. spp.) with more than 100 described (Edel‐Hermann and Lecomte 2019) and these may be further subdivided into races based on their ability to infect and cause symptoms on particular sets of different host lines, varieties or cultivars (Leslie and Summerell 2006). More recently however, the traditional model of host specificity and the concept of ff. spp. in *Fusarium* pathogens is being challenged in favour of a shift of perspective to a “pathobiome” outlook, where disease dynamics are driven by community‐level interactions (Han et al. 2025).

Lettuce ( *Lactuca sativa* ) is an important leaf crop globally with nearly 27 Mt of lettuce (and chicory) produced from 1.2 million hectares worldwide, yielding approximately 21.9 t/ha (FAOSTAT 2023). However, lettuce production is increasingly affected by Fusarium wilt disease caused by *F. oxysporum* f. sp. *lactucae* (Fola), which results in symptoms of stunting, leaf chlorosis and plant death, with crop losses of > 50% reported (Gilardi, Franco Ortega, et al. 2017; Gilardi, Pons, et al. 2017). Fola was first identified in Japan in 1967 (Matuo and Motohashi 1967) and has since been found in multiple lettuce‐producing countries worldwide. Four races (Fola1, Fola2, Fola3 and Fola4) have been identified so far with Fola1 being the most predominant globally (Cabral et al. 2014; Garibaldi et al. 2002; Huang and Lo 1998; Hubbard and Gerik 1993; Malbrán et al. 2014; van Amsterdam et al. 2023). Fola2 and Fola3 have been identified only in Asia (Fujinaga et al. 2005; Lin et al. 2014) while Fola4 has only emerged relatively recently having been first identified in the Netherlands in 2013 (Gilardi, Franco Ortega, et al. 2017). Since then, Fola4 has spread through different parts of Europe with reports from Belgium (Claerbout et al. 2017), the UK (Taylor et al. 2018), Italy (Gilardi et al. 2019) and Spain (Gálvez et al. 2023). Fola4 initially only affected protected lettuce but has now been reported in open field production in Italy and Spain (Gálvez et al. 2023; Gilardi et al. 2019). More recently, Fola variants have been reported that overcome known sources of resistance to Fola1 (Nayak et al. 2025) and Fola4 (Mestdagh et al. 2025). Phylogenetically, Fola4 has been found to be very close to Fola1 with both races clustering with the species *Fusarium curvatum* (Waqas et al. 2025).

Plant pathogens have evolved mechanisms to overcome host resistance including through the secretion of effectors, that can alter and modulate plant processes in order to suppress plant defences (Win et al. 2012). In *F. oxysporum*, the Secreted in Xylem (Six) proteins encoded by *SIX* genes are the most extensively studied putative effectors (Rep et al. 2004; Takken and Rep 2010) and were first identified following xylem sap proteomic analyses of tomato plants infected with *F. oxysporum* f. sp. *lycopersici* (Foly) (Houterman et al. 2007; Lievens et al. 2009; Schmidt et al. 2013). Fourteen Six proteins have been identified so far, with Six1 (Rep et al. 2005), Six3 (Houterman et al. 2009), Six6 (Gawehns et al. 2014) and Six5 (Ma et al. 2015) shown to contribute to virulence on tomato and characterised as effector proteins. Homologues of *SIX* genes have since been identified in a wide range of *F. oxysporum* ff. spp. (Jangir et al. 2021), and are often located on accessory chromosomes alongside other effector candidates (Bates et al. 2024; Ma et al. 2010; Yang et al. 2020).

Proof of the role of effector genes in virulence is often determined through gene knockout studies, as deletion/disruption of genes through reverse genetic approaches is an effective means of beginning to understand their function (Wang et al. 2018). Different systems have been developed for the targeted knockout of genes in filamentous fungi, such as protoplast‐mediated transformation (PMT), *Agrobacterium*‐mediated transformation (AMT) and more recently CRISPR (clustered regularly interspaced short palindromic repeats)‐Cas9 (CRISPR‐associated protein 9) systems (Gardiner and Kazan 2018; Li et al. 2017; Michielse et al. 2005; Tilburn et al. 1983). Notably, the first documented CRISPR‐Cas9 transformation of *F. oxysporum* was carried out using in vitro‐prepared sgRNA/Cas9 ribonucleoprotein (RNP) complexes where *URA3* (encoding orotidine 5′‐phosphate decarboxylase) and *URA5* (encoding orotate phosphoribosyltransferase) genes in *F. oxysporum* f. sp. *vasinfectum* (Fov) were successfully knocked out (Wang et al. 2018). *SIX1* (*AVR3*), *SIX4* (*AVR1*), *SIX3* (*AVR2*), *SIX5* and *SIX6* in Foly have since been investigated by knockout and complementation of each respective gene through AMT (Gawehns et al. 2014; Houterman et al. 2008, 2009; Ma et al. 2015; Rep et al. 2004). *SIX* gene functional studies in other *F. oxysporum* ff. spp. have also been carried out, such as in *F. oxysporum* f. sp. *niveum* (Niu et al. 2016), *F. oxysporum* f. sp. *radicis‐cucumerinum* (van Dam et al. 2017), *F. oxysporum* f. sp. *cubense* (Focub) (An et al. 2019; Widinugraheni et al. 2018), *F. oxysporum* f. sp. *conglutinans* (Focn) (Ayukawa et al. 2021) and in *F. oxysporum* f. sp. *cepae* (Sakane et al. 2023). Recently, a vector based CRISPR‐Cas9 system was described for the knockout of *SIX1* in Foly with a reported transformation efficiency of 86%, demonstrating effective CRISPR‐Cas9 genome editing of in *F. oxysporum* (Shinkado et al. 2022). Shinkado et al. (2022) also investigated the efficiency of their CRISPR‐Cas9 vector system through homology‐directed repair (HDR)‐mediated targeted gene knockout of the Ku80, Ku70 and Lig4 genes involved in non‐homologous end‐joining (NHEJ)‐mediated repair (Kito et al. 2008; Ninomiya et al. 2004). Here, their optimised CRISPR‐Cas9 system increased transformation efficiency from 42% to 50% (using a standard PMT approach) to 100% for all three genes (Shinkado et al. 2022), thereby highlighting CRISPR‐Cas9 systems can be highly efficient for targeted gene knockout studies in *F. oxysporum*.

To date, there have been no functional studies investigating the role of *SIX* genes or other putative effectors in the virulence of Fola races on lettuce. Fola4 and Fola1 display different virulence profiles on the lettuce differential set whereby Fola4 breaks sources of Fola1 resistance (e.g., cv. Costa Rica; ISF 2022). Analysis of Fola1 and Fola4 genomes and PCR‐based isolate profiling has determined that both races harbour homologues of *SIX9* and *SIX14*, while Fola4 isolates additionally contain *SIX8,* which is divergently transcribed with *Pair with Six Eight 1* (*PSE1*) or *Pair with Six Eight 1*‐ *Like* (*PSL1*) (Bates et al. 2024; Legg 2024). *SIX8* is of particular interest as it has been implicated in the virulence of Foly on tomato, Focn on *Arabidopsis* and Focub Tropical Race 4 (Focub TR4) on banana (Aalders et al. 2024; An et al. 2019; Ayukawa et al. 2021). Cavendish banana cultivars, which are resistant to Focub race 1 (Focub1), are susceptible to Focub TR4. Among the *SIX* genes identified in Focub1 and Focub TR4, *SIX2* and *SIX8* are only detected in Focub TR4 and not identified in Focub1. Work by An et al. (2019) reported *SIX8* to be required for virulence of Focub TR4 on Cavendish banana plantlets. *SIX8* is therefore potentially an important effector in Fola4 and is present as a single copy in the genome, which facilitates gene knockout studies. This research aimed to examine the role of *SIX8* in Fola4 virulence through generation and phenotyping of CRISPR‐Cas9‐mediated knockout and *Agrobacterium*‐mediated complementation mutants.

## 2 Results

### 2.1 Fola4 *SIX* Gene Expression Increases During Early Stages of Lettuce Infection

**FIGURE 1:**
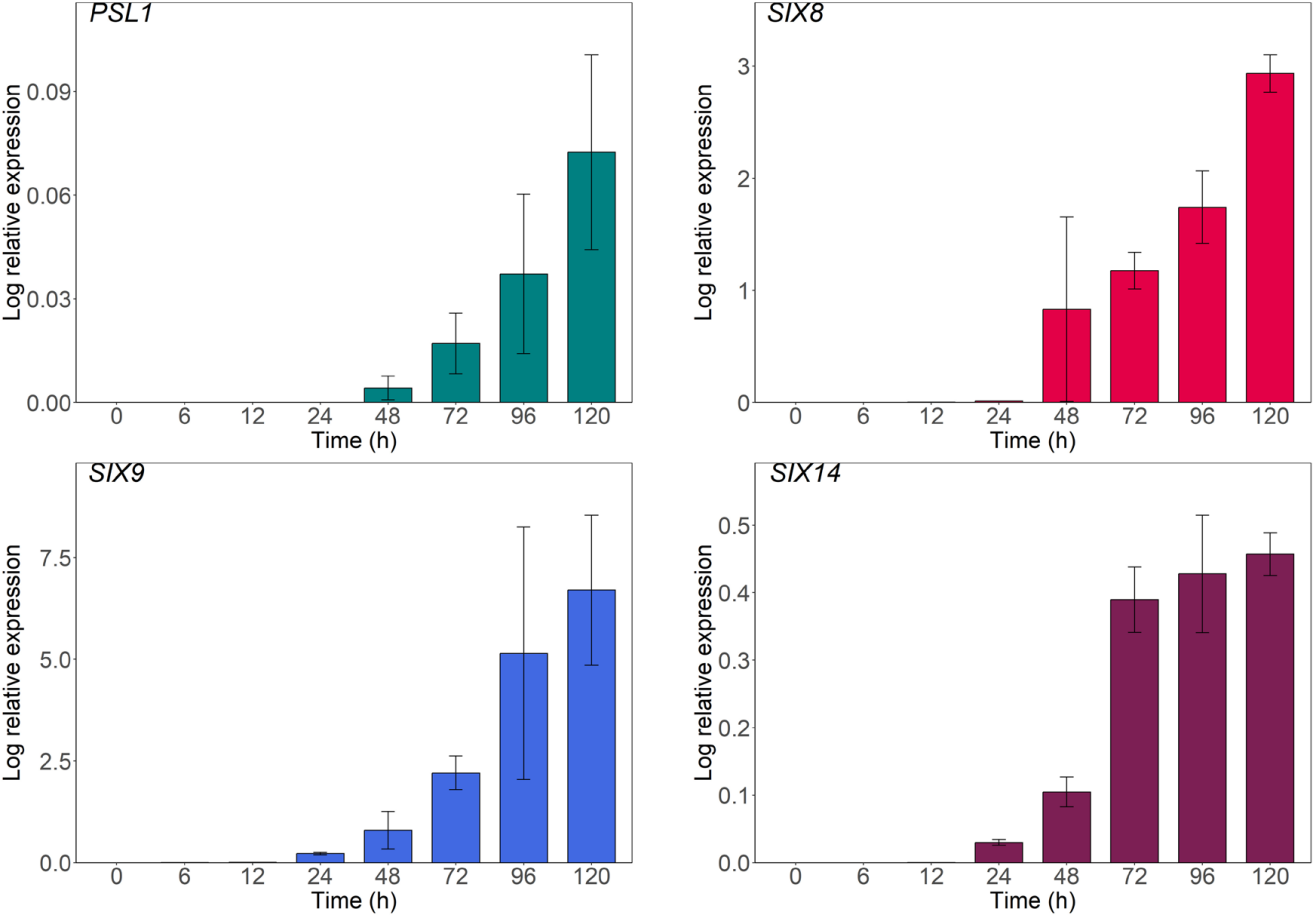
Expression of *Secreted In Xylem* (*SIX*) genes and *PSL1* over time as determined by reverse transcription‐quantitative PCR in roots of lettuce cv. Steamboat following infection with *Fusarium oxysporum* f. sp. *lactucae* race 4 (Fola4) isolate AJ516. Expression values were calculated relative to *Translation Elongation Factor 1a* (*TEF*). Values represent means of relative gene expression values from four replicate plates of 10 lettuce seedlings at eight time points (0–120 h) post‐inoculation.

The expression of Fola4 *SIX* genes (*SIX8*, *SIX9*, *SIX14*) and other putative effectors following lettuce infection was previously established through RNA‐seq analysis 96 h post‐inoculation (hpi) using an in vitro seedling bioassay on agar plates (Bates et al. 2024). To further confirm the potential importance of *SIX8* and other *SIX* genes in infection, the relative expression of *SIX8*, *SIX9*, *SIX14* and *PSL1* (transcribed with *SIX8*) over time (0–120 hpi) was examined using reverse transcription‐quantitative PCR (RT‐qPCR) assays following inoculation of lettuce seedlings (cv. Steamboat) with Fola4 isolate AJ516 using the same system. This showed significant increases in log relative expression values for all three *SIX* genes and *PSL1* between 48 and 96 hpi (Figure 1, Table S1) suggesting a potential role in infection. Relative expression values also varied significantly between these genes with *SIX9* expression the highest followed by *SIX8*, *SIX14* and then *PSL1* at 120 hpi (Table S1). Moreover, initial detection of expression also varied with *SIX8* and *PSL1* detected later than *SIX9* and *SIX14* (24 and 48 hpi for *SIX8* and *PSL1*, respectively, compared to 12 hpi for *SIX9* and *SIX14*).

### 2.2 Fola4 *SIX8* Knockouts Show Reduced Virulence on Lettuce Seedlings

**FIGURE 2:**
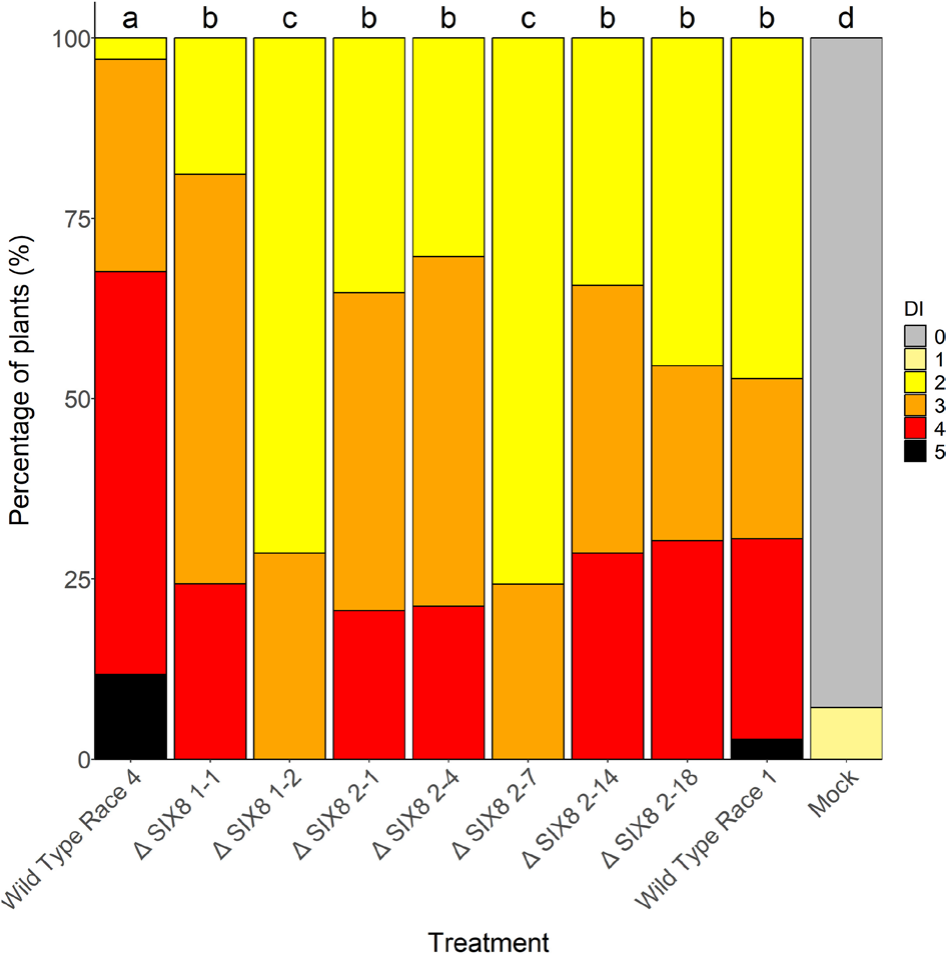
Root browning disease symptom scores (DI) after 28 days following inoculation of lettuce seedlings (cv. Steamboat) in vitro with seven *Fusarium oxysporum* f. sp. *lactucae* race 4 (Fola4) AJ516 *SIX8* knockout mutants, wild type Fola4 isolate AJ516 and Fola1 isolate AJ520. Groups denoted by the same letter indicate no significant difference as determined by the Kruskal–Wallis test followed by Dunn's post hoc analysis with Benjamini–Hochberg adjustment at the 5% significance level.

To investigate the role of *SIX8* in Fola4 infection, *SIX8* knockout mutants of isolate AJ516 were generated using a CRISPR‐Cas9‐mediated system and seven of these (with similar growth to wild type [WT]) were evaluated for virulence using an in vitro lettuce seedling bioassay (cv. Steamboat). Comparisons of disease score categories (based on severity of root browning symptoms) were made with WT Fola4 AJ516, WT Fola1 isolate AJ520, and an uninoculated (mock) control. Root browning was reduced for all the Fola4 *SIX8* knockout mutants at each of four disease assessment timepoints (Figure S1a) compared with WT Fola4 AJ516, and symptoms were restricted to lateral roots with less browning observed in the main tap root (Figure S1c). Dunn's post hoc tests at the final 28 days post‐inoculation (dpi) timepoint revealed root browning was significantly reduced for all the *SIX8* knockout mutants compared to WT and was comparable with the Fola1 AJ520 isolate (Figure 2, Table S2a).

### 2.3 Complementation of *SIX8* in a Fola4 Knockout Mutant Restores Virulence in Lettuce Seedlings

**FIGURE 3:**
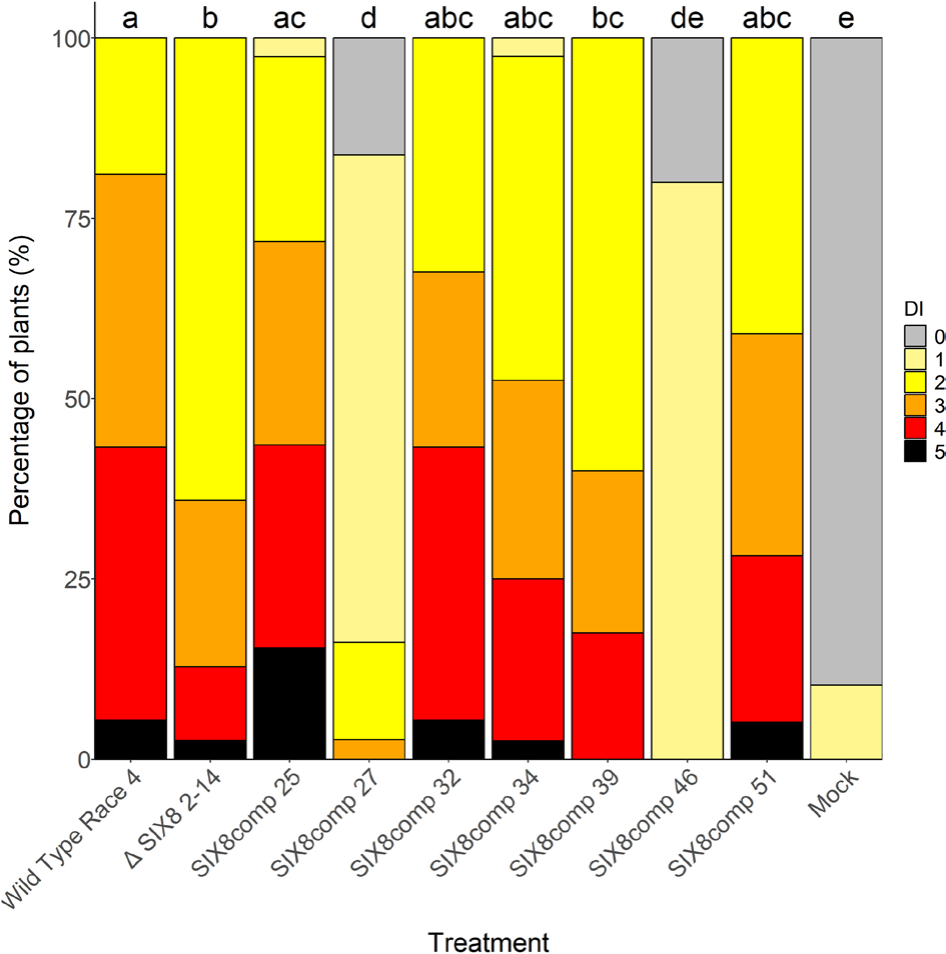
Root browning disease symptom scores (DI) after 28 days following inoculation of lettuce seedlings (cv. Steamboat) in vitro for seven *Fusarium oxysporum* f. sp. *lactucae* race 4 (Fola4) *SIX8* complementation mutants, wild‐type Fola4 isolate AJ516, and *SIX8* knockout mutant ΔSIX8 2‐14. Groups denoted by the same letter indicate no significant difference as determined by the Kruskal–Wallis test followed by Dunn's post hoc analysis with Benjamini–Hochberg adjustment at the 5% significance level.

To further confirm the role of *SIX8* in Fola4 infection, Fola4 *SIX8* complementation mutants of ΔSIX8 2–14 were generated using an *Agrobacterium‐*mediated system and seven tested for virulence in a second in vitro lettuce seedling bioassay (cv. Steamboat). Comparisons in root browning disease score were made with WT Fola4 AJ516, ΔSIX8 2‐14 and the uninoculated (mock) control. Root browning was comparable to WT Fola4 for four complementation mutants (SIX8comp25, SIX8comp32, SIX8comp34 and SIX8comp51) at each of four disease assessment timepoints (Figure S1b), and did not differ significantly from WT Fola4 at 28 dpi, indicating restoration of function (Figure 3). Furthermore, these complementation mutants resulted in more severe root browning symptoms at 28 dpi compared to ΔSIX8 2‐14 (Figure 3) although this was only significant for SIX8comp25 (Table S2b). Two *SIX8* complementation mutants (SIX8comp27, SIX8comp46) resulted in significantly reduced root browning scores compared to WT Fola4 but were subsequently found to have restricted growth on potato dextrose agar (PDA), most likely due to deleterious effects of *Agrobacterium‐*mediated transformation (Figure 3, Figure S2c).

### 2.4 Fola4 Knockout and Complementation Mutants Show Reduced and Restored Virulence in Glasshouse Grown Lettuce

**FIGURE 4:**
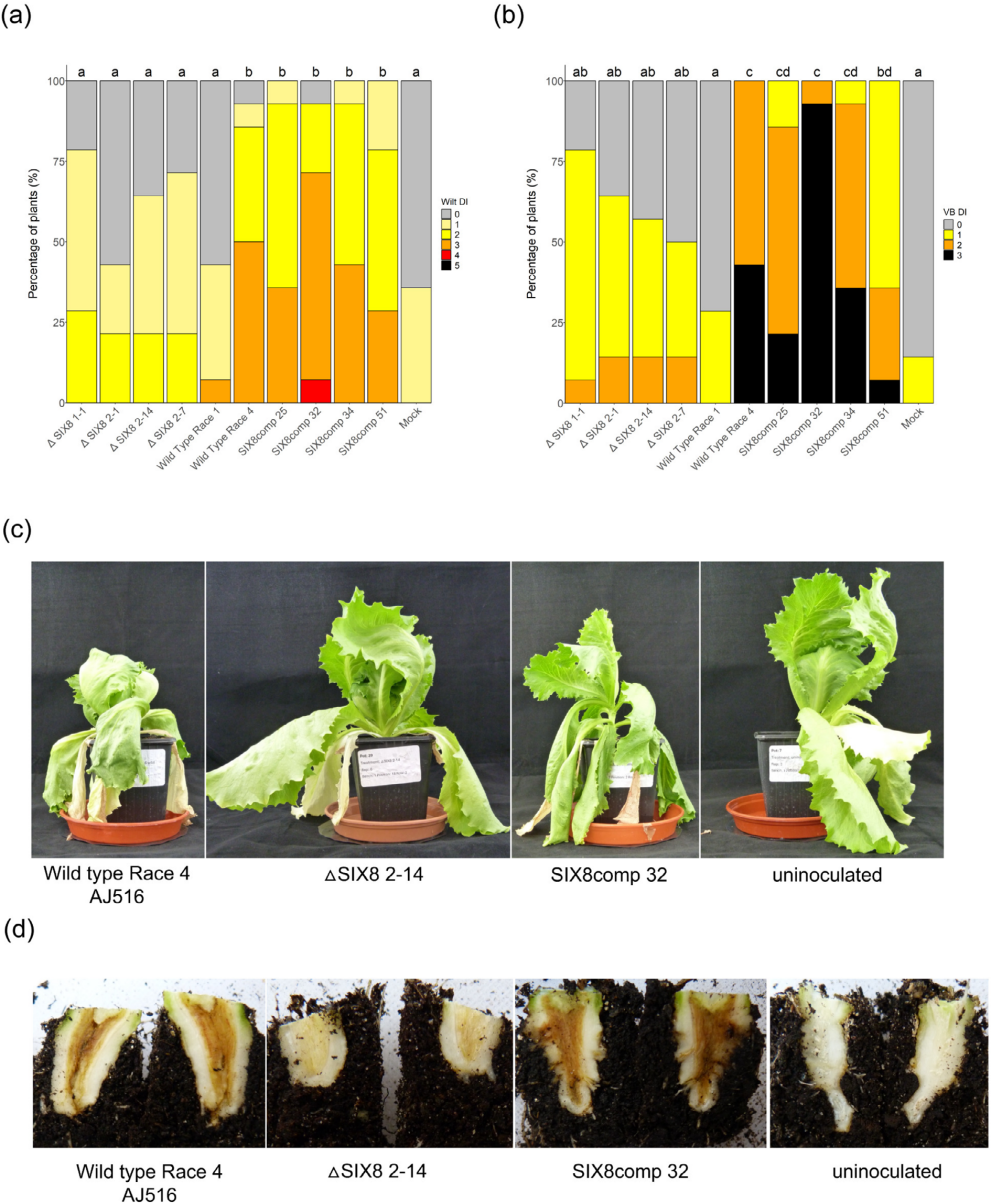
(a) Wilt disease scores (Wilt DI) and (b) vascular browning scores (VB DI) after 38 days and 42 days, respectively, following inoculation of lettuce plants (cv. Steamboat) with four *Fusarium oxysporum* f. sp. *lactucae* race 4 (Fola4) *SIX8* knockout mutants, four *SIX8* complementation mutants, wild type Fola4 isolate AJ516 and Fola1 isolate AJ520. Groups denoted by the same letter indicate no significant difference as determined by the Kruskal–Wallis test followed by Dunn's post hoc analysis with Benjamini–Hochberg adjustment at the 5% significance level. (c) Wilt and (d) vascular browning symptoms following inoculation of lettuce (cv. Steamboat) with wild type Fola4 AJ516, Fola4 *SIX8* knockout mutant ΔSIX8 2‐14 and Fola4 *SIX8* complementation mutant SIX8comp32 compared with an uninoculated control.

The virulence of four of the Fola4 knockout mutants (ΔSIX8 1‐1, ΔSIX8 2‐1, ΔSIX8 2‐7, ΔSIX8 2‐14) and four of the complementation mutants (SIX8comp25, SIX8comp32, SIX8comp34, SIX8comp51—all derived from ΔSIX8 2–14) was also evaluated on lettuce cv. Steamboat in a pot‐based glasshouse experiment and compared with WT Fola4 AJ516, WT Fola1 AJ520 and an uninoculated (mock) control. All four Fola4 *SIX8* knockout mutants resulted in significantly reduced lettuce wilt disease scores compared with WT Fola4 at 38 dpi (Figure 4a,c, Figure S3a) as well as vascular browning score following tap root dissection at 42 dpi (Figure 4b,d). WT Fola1 isolate AJ520 also resulted in significantly lower wilt score (Figure 4a, Table S3a) and vascular browning score (Figure 4b, Table S3b) compared to WT Fola4 and did not differ significantly from any of the *SIX8* knockout mutants. Infection of lettuce with three of the *SIX8* complementation mutants (SIX8comp25, SIX8comp32, SIX8comp34) resulted in comparable disease symptoms to WT Fola4 with no significant differences in wilt disease score at 38 dpi (Figure 4a, Table S3a) or root vascular browning at 42 dpi (Figure 4b, Table S3b). However, SIX8comp51 had significantly reduced virulence compared with WT Fola4 (Figure 4b, Table S3b). Complementation mutants SIX8comp25, SIX8comp32, SIX8comp34 also resulted in significantly greater wilt disease and root vascular browning scores compared with the Fola4 *SIX8* knockout mutants and uninoculated control treatment after 38 and 42 dpi, respectively (Figure 4a,b, Table S3a,b). Interestingly, the *SIX8* complementation mutant SIX8comp32 resulted in greater wilt disease than the Fola4 wild type control from 13 dpi onwards, although this was not significantly different at 38 dpi (Figure 4a, Figure S23a). Additionally, mean dry weights of WT Fola4 and complementation mutant treated plants significantly differed from the uninoculated control which was not the case for plants treated with knockout mutants and WT Fola1 (Figure S3b, Table S3c). WT Fola4 and complementation mutants resulted in lower mean dry weights compared to knockout mutants and WT Fola1 although not all pairwise comparisons showed significant differences (Figure S3b, Table S3c).

## 3 Discussion

The first part of this study demonstrated that expression of putative Fola effectors *SIX8*, *SIX9*, *SIX14* and *PSL1* following infection of lettuce seedlings increased over time up to 96 h (4 days) with no significant increase observed at 120 hpi. Moreover, it was observed that the four genes had different levels of expression with *SIX9* showing the highest levels. Fola4 isolate AJ516 contains four copies of *SIX9* (*SIX9.2*, *SIX9.3* and two copies of *SIX9.4*; Bates et al. 2024) and the qPCR primers designed here amplify the two *SIX9.4* copies. This could therefore explain the significantly greater levels of expression in *SIX9* compared to the single copies of *SIX8*, *SIX14* and *PSL1* present in Fola4 AJ516. *In planta SIX* gene RT‐qPCR expression investigations in other *F. oxysporum* ff. spp. have reported similar expression profiles over time. For example, Taylor et al. (2016) reported significant increases in the expression of *SIX3*, *SIX5*, *SIX7*, *SIX9*, *SIX10* and *SIX12* between 36 and 72 hpi in *F. oxysporum* f. sp. *cepae* (isolate FUS2) but expression levels then generally decreased from 72 to 96 hpi. However, Jenkins et al. (2021) reported significant increases in the expression of *SIX7*, *SIX10*, *SIX11* and *SIX12* between 72 and 96 hpi in *F. oxysporum* f. sp. *pisi* (isolate FOP1 EMR), which better matches the results found here for Fola4, although no further timepoints were investigated. Additionally, Thatcher et al. (2016) and Williams et al. (2016) reported significant increases in *SIX1*, *SIX8*, *SIX9* and *SIX13* expression over the course of 7 days in *F. oxysporum* f. sp. *medicaginis* (isolate 5190a), while Sun et al. (2022) observed significant increases in *SIX9* expression over the course of 6 days in Foly (isolate 007).

The main results of this research demonstrate a role for *SIX8* in Fola4 virulence on lettuce for the first time using knockout and complementation mutants generated by CRISPR‐Cas9 and AMT approaches, respectively. A reduction in virulence was observed for all Fola4 *SIX8* knockout mutants in both in vitro and glasshouse lettuce bioassays, but complete loss of virulence was not observed, indicating that additional genes contribute to Fusarium wilt disease in lettuce. This is not surprising as Fola1 lacks the *SIX8* gene yet is still pathogenic on certain lettuce cultivars. Interestingly, Fola1 AJ520 generally resulted in disease scores similar to the Fola4 *SIX8* knockout mutants in both in vitro and glasshouse experiments, suggesting that the gain of *SIX8* by Fola4 may have resulted in increased virulence. However, our phenotyping was only carried out using one lettuce cv. (Steamboat), which, although considered to be susceptible to both Fola1 and Fola4, may have some partial resistance to the former. It could also be hypothesised that *SIX8* allows Fola4 to overcome some sources of Fola1 resistance and hence infect a different range of lettuce cvs, as reported previously (ISF 2022). Future work could therefore determine the virulence of the *SIX8* knockout and complementation mutants against a larger panel of lettuce cultivars with different resistance profiles to better elucidate the effect of *SIX8* on host cultivar range. Reduction in virulence by knocking out *SIX8* has also been observed for other *F. oxysporum* ff. spp. For instance, a *SIX8* knockout mutant of Focub TR4 resulted in reduced virulence on banana compared with the wild‐type isolate, but, as observed here for Fola4, did not completely abolish disease (An et al. 2019). This was also found to be the case for Focn *SIX8*‐*PSE1* knockout mutants, which were also less virulent on *Arabidopsis* plants compared with wild‐type Focn (Ayukawa et al. 2021).

The role of Six8 in both Foly and Fo strain Fo5176 (Foa) causing wilt disease in *Arabidopsis thaliana* has been postulated to occur through interaction with plant transcriptional corepressors in the TOPLESS (TPL) family (Aalders et al. 2024). Aalders et al. (2024) demonstrated that gene knockouts of *TPL1* and *TPL2* as well as *AtTPL* and *AtTPR1* in tomato and *Arabidopsis* reduced virulence of Foly and Foa, respectively. Future studies could therefore investigate interactions of Fola4 Six8 with respective Tpl homologues in lettuce. Lettuce has 10 *TPL*/*TPR* genes according to genomic information from *Lactuca sativa* ‘Salinas’, NCBI accession: GCF_002870075.4 with two *TPL1* and one *TPL2* homologues, respectively (Legg 2024). Phylogenetic analysis (unpublished) has indicated that protein sequences of the lettuce Tpl1 and Tpl2 homologues shared high homology with those in tomato and *Arabidopsis*. Aalders et al. (2024) demonstrated that Foa Six8 interacts with the first WD40 domain of AtTpr1, and therefore it would be interesting to investigate if Fola4 Six8 also interacts with the lettuce Tpl1/Tpl2 WD40 domains. Ayukawa et al. (2021) reported that both *SIX8* and *PSE1* are needed for full virulence of Focn on *Arabidopsis* and that a *PSE1‐Like* (*PSL1*) gene was present in the Foly genome, although the roles of *SIX8* and *PSL1* in Foly virulence on tomato have yet to be investigated. Functional differences between *PSE1* and *PSL1* were also reported by Ayukawa et al. (2021), such that complementation of Focn mutants lacking *SIX8*‐*PSE1* with Foly *SIX8*‐*PSL1* did not result in restored virulence on *Arabidopsis*, while complementation using *SIX8‐PSE1* from the related crucifer‐infecting *F. oxysporum* f. sp. *matthiolae* resulted in disease. Interestingly, there are two sequence variants of *SIX8* present within Fola4 that are paired with homologues of either *PSE1* or *PSL1* in each isolate (Bates et al. 2024; Legg 2024). However, functional differences between these Fola4 variants such as lettuce cultivar host range have yet to be examined and it is also unclear whether Fola4 virulence on lettuce requires both *SIX8* and either *PSE1* or *PSL1*. Physical interactions between the Foly Six8 and Psl1 proteins have recently been confirmed, such that they form a heterodimer through inter‐disulphide bonds between free cysteine residues present in the two protein complexes and those residues were also found to be conserved in Focn Six8 and Pse1 (Yu et al. 2024). Fola4 *SIX8*‐*PSE1*/*PSL1* gene homologues were found to be very similar to *PSE1*/*PSL1* homologues from Focn and Foly, respectively (Legg 2024), and hence further work could investigate Fola4 Six8‐Pse1/Psl1 protein interactions during infection of lettuce.

Although the gain of *SIX8* by Fola4 isolates could be involved in the differential virulence of Fola1 and Fola4 on different lettuce cultivars, other effectors may also be involved. Bates et al. (2024) reported additional Fola race‐specific effectors expressed *in planta*, highlighting three that were Fola1‐specific and eight Fola4‐specific (including *SIX8*), while two effectors were common to both Fola1 and Fola4 with no significant homology to effectors from other *F. oxysporum* ff. spp. These additional effectors may act as race‐specific virulence factors or potential avirulence genes. Knockout studies of Fola‐specific and race‐specific effector genes in Fola1 and Fola4 alongside plant infection bioassays on susceptible and resistant lettuce material may provide additional insights into virulence and avirulence in Fola as has been elucidated in the Foly pathosystem (Gawehns et al. 2014; Houterman et al. 2008, 2009; Rep et al. 2004). Little is known about the nature of Fola resistance genes in lettuce, although quantitative trait loci (QTLs) have been reported for Fola1 resistance (Murray et al. 2021; Seki et al. 2020). Specific genes responsible for Fola resistance have yet to be determined, and, as such, plant resistance (*R*) gene/effector interactions are still to be uncovered.

## 4 Experimental Procedures

### 4.1 Fungal Strains, Plant Materials and Bioassays on Lettuce

The Fola4 and Fola1 susceptible lettuce cv. Steamboat used in this study was provided by Enza Zaden (Netherlands). The Fola4 isolate AJ516 was originally isolated from diseased lettuce from Lancashire, UK (Taylor et al. 2018). The Fola1 isolate AJ520 (MYA 3040) also included in phenotyping was from diseased lettuce from Italy (Garibaldi et al. 2002).

### 4.2 Fola4 *SIX* Gene Expression During Early Stages of Lettuce Infection

Experiments were set up to investigate Fola4 *SIX* gene expression over time during lettuce infection using RT‐qPCR.

#### 4.2.1 Inoculation of Lettuce Seedlings With Fola4 Using an In Vitro System

Lettuce seeds were surface sterilised by immersing in FICHLOR solution (3.1 g sodium dichloroisocyanurate dihydrate and one drop of Nonidet in 50 mL deionised water) followed by gentle shaking at approximately 200 rpm for 4 min. Seeds were then rinsed in sterile deionised water (SDW) and 10 seeds placed across individual square Petri dish plates (12 × 12 × 1.7 cm, Greiner Bio‐One) containing autoclaved ATS medium (1 M KNO_3_, 1 M KPO_4_, 1 M MgSO_4_, 1 M Ca(NO_3_)_2_, 20 mM Fe‐EDTA, 70 mM H_3_BO_3_, 14 mM MnCl_2_, 0.5 mM CuSO_4_, 1 mM ZnSO_4_, 0.2 mM Na_2_MoO_4_, 10 mM NaCl, 0.01 mM CoCl_2_, 0.45% Gelrite [Duchefa Biochemie]). Plates were sealed with tape and cling‐filmed in stacks after which they were incubated at 4°C in the dark for 4 days, then at 15°C in light/dark (16 h daylength) for 7 days, and finally at 25°C in light/dark (16 h daylength) for 5 days to promote seedling growth until the main tap roots began to reach the end of the plate.

Fola4 isolate AJ516 was grown on PDA at 20°C for approximately 14 days to produce spore suspensions. Spore suspensions were prepared by adding 20 mL of SDW to PDA cultures and using a sterile spreader to release spores. The suspension was then filtered through three layers of Miracloth to filter out mycelium. The concentration of the resulting spore suspension was adjusted to 10^6^ spores mL^−1^ with 0.012% Tween 20 in SDW.

The Fola4 AJ516 spore suspension was used for inoculation, with 1.5 mL at a concentration of 10^6^ spores mL^−1^ pipetted directly over the lettuce seedling roots in each plate with tilting from left to right to distribute the inoculum evenly. Plates were then allowed to dry under sterile airflow for approximately 15 min before sealing with tape. Plates were wrapped in clingfilm in timepoint batches (four replicates per timepoint), after which they were then placed in a randomised block design in an incubator at 25°C (16 h photoperiod). Plate packs were removed at 6, 12, 24, 48, 72, 96 and 120 hpi with the 0 h timepoint (pre‐inoculation) and a negative control (roots inoculated with SDW + Tween and harvested at 96 hpi) also included. At each timepoint, the roots of all 10 lettuce plants for each of the four plates were removed, rinsed in SDW, flash frozen in liquid nitrogen and stored at −80°C.

#### 4.2.2 RNA Extraction and cDNA Synthesis

Lettuce roots were ground to a fine powder using a pestle and mortar filled with liquid nitrogen and approximately 100 mg of tissue was transferred to a 2 mL tube. This frozen root material was then ground further using a Dremel drill (model 398, with a rounded drill bit) and then RNA extracted using TRIzol reagent (Ambion, Thermo Fisher Scientific) following the manufacturers' guidelines. Extracted RNA was precipitated using 900 μL of lithium chloride to 100 μL RNA (250 μL LiCl_2_ + 650 μL DEPC‐treated water) to remove contaminants. Remaining DNA was removed from samples using DNase I (Sigma‐Aldrich). RNA samples were visualised on a 2% agarose gel (containing GelRed at 2 μL per 100 mL of gel) with the addition of loading dye (Orange G, Sigma‐ Aldrich), to check for degradation. First‐strand cDNA was synthesised using Superscript II reverse transcriptase (Invitrogen) following the manufacturer's protocol.

#### 4.2.3 Quantifying *SIX* Gene Expression Using RT‐qPCR

The expression of *SIX8*, *SIX9*, *SIX14* and *PSL1* genes relative to *Translation Elongation Factor 1α* (*TEF*) identified in Fola4 was assessed using qPCR of the cDNA produced from each of the inoculated lettuce root samples at each time point. Primers for each *SIX* gene were designed within the coding region using Primer3Plus (Untergasser et al. 2007) or by manually selecting candidate primers (Table S4). Primers were checked for self‐hybridisation and secondary DNA structure formation using the OligoAnalyser tool (Integrated DNA Technologies Inc.). qPCR was performed in a QuantStudio 5 Real‐Time PCR machine using PowerUp SYBR Green Master Mix (Applied Biosystems, Thermo Fisher Scientific), following the manufacturers' protocol. All primers were used at a final concentration of 0.5 μM (except *TEF* primers which were used at 0.4 μM) in a final reaction volume of 19 μL per well, using the following conditions: one cycle of 95°C for 120 s; 45 cycles of 95°C for 3 s, primer annealing temp (Table S4) for 30 s. A melt curve analysis (following amplification) was used to confirm the presence of a single PCR product. All samples were run in triplicate, standard curves were prepared for each gene target, by using serially diluted genomic Fola4 DNA, and for analysis, the cDNA concentration of each gene expressed relative to *TEF*. All *SIX* gene qPCRs also included a negative control (roots inoculated with SDW + Tween 20 and harvested at 96 hpi), but no *SIX* gene expression was observed in any of these samples (data not shown).

### 4.3 Generation of Gene Knockout and Complementation Constructs

#### 4.3.1 Design and Synthesis of *SIX8* Specific Single Guide RNAs

The Fola4 isolate AJ516 *SIX8* gene sequence was imported into Geneious Prime (2023.0.4, Build 2023‐01‐24) and the CRISPR site prediction tool used to identify sites with protospacer and PAM sequences of (N)_20_NGG in the *SIX8* coding region. Candidate sgRNAs were then sorted based on their activity score (Doench et al. 2016), specificity score (Ran et al. 2013) and proximity to the 5′ end of the gene coding region. Off‐targets were scored against the Fola4 AJ516 genome assembly and only sgRNAs that were 100% specific selected. Two *SIX8* specific sgRNAs were selected for generation and testing by in vitro *SIX8* DNA cleavage. *SIX8* sgRNA target oligomers (T7 promoter‐(N)_20_‐GTTTTAGAGCTAGAAATAGCAAG) and an sgRNA universal oligomer (Table S5) were used to generate the two *SIX8* sgRNAs (Table S6) using the EnGen sgRNA synthesis kit (New England Biolabs).

#### 4.3.2 Design and Cloning of Donor Fola4 *SIX8* Knockout Plasmid

Construction of a plasmid for use in the CRISPR‐Cas9‐mediated knockout of *SIX8* was carried out using NEBuilder HiFi DNA Assembly Master Mix Kit (New England Biolabs). *SIX8* flanking regions were amplified from Fola4 AJ516 genomic DNA using HiFi primers (Table S5) and assembled into the pPK2HPHGFP plasmid (Michielse et al. 2009) either side of a selection cassette containing hygromycin phosphotransferase (*hph*) and green fluorescent protein (*gfp*) genes. PCR amplification was then carried out on the cloned plasmid (pPK2HPHGFP_CRISPR_SIX8; Figure S4a) to produce the final donor DNA template using primers (*SIX8* Left flank fwd and *SIX8* Right flank rev) listed in Table S5 and outlined in Figure S4a.

#### 4.3.3 Design and Cloning of *Agrobacterium*‐Mediated Transformation Plasmid for the Complementation of a Fola4 *SIX8* Knockout Mutant

Construction of a plasmid for *SIX8* complementation was carried out using the NEBuilder HiFi DNA Assembly Master Mix Kit (New England Biolabs). In this approach, the left flank was composed of the *SIX8* putative promoter (1 kb region upstream of *SIX8*), the *SIX8* gene and the *SIX8* putative terminator (500 bp region downstream of *SIX8*; Figure S4b). The region 559 bp downstream of the putative *SIX8* terminator was selected as the right flank (Figure S4b). Flanking regions were amplified from Fola4 genomic DNA using HiFi primers (Table S5) and assembled into the PRW1p plasmid (Houterman et al. 2008) either side of a selection cassette containing a zeocin resistance (*ble*) gene. The plasmid was then transformed into *A. tumefaciens* EHA105 using a freeze‐thaw transformation approach (Chen et al. 1994).

### 4.4 Fungal Transformation

#### 4.4.1 CRISPR Cas9 Mediated Knockout of *SIX8*

Protoplast isolation was performed using a protocol based on methods previously described (Brückner et al. 1992; Janevska et al. 2018; Tudzynski et al. 1999; Vlieger et al. 2025). Precultures of Fola4 isolate AJ516 were initiated by inoculation of Darken medium (15 g L^−1^ cornsteep solids, 30 g L^−1^ sucrose, 1 g L^−1^ (NH_4_)_2_SO_4_, 7 g L^−1^ CaCO_3_) and incubated at 200 rpm for 48–72 h at 25°C. Precultures (500 μL) were then used to generate main Fola4 AJ516 cultures by inoculation of ICL medium (80 g L^−1^ D‐glucose, 1 g L^−1^ MgSO_4_, 0.5 g L^−1^ KH_2_PO_4_, 2 mL L^−1^ trace elements solution, 6 mM glutamine). Main cultures were incubated in an orbital shaker at 200 rpm for 17 h at 25°C, after which fungal germlings were collected, washed and treated with a mixture of 4 mg mL^−1^ lysing enzyme mix (Sigma‐Aldrich), 0.2 mg mL^−1^ lyticase (Sigma), 0.2 mg mL^−1^ yatalase (TakaraBio) and 0.2 mg mL^−1^ bovine serum albumen (BSA) suspended in 1.2 M KCl, 50 μM CaCl_2_ for 3–4 h at 28°C with shaking at 90 rpm. Fola4 protoplasts were then collected, separated from remaining fungal mycelium by filtering through Miracloth, and the concentration adjusted to 2 × 10^7^ protoplasts mL^−1^ in STC buffer (1.2 M sorbitol, 0.01 M Tris–HCl pH 7.5, 0.05 M CaCl_2_).

*Fusarium oxysporum* protoplast transformation was performed using a protocol based on methods previously described (Brückner et al. 1992; Janevska et al. 2018; Pokhrel et al. 2022; Tudzynski et al. 1999; Vlieger et al. 2025). Cas9 RNPs were assembled in a 1:1 mol ratio of Cas9:sgRNA into a final volume of 50 μL composed of 20 μg Cas9, 20 μg sgRNA, 5 μL 10× Cas9 nuclease buffer, and DEPC‐treated water added to volume. The mixture was then incubated at 37°C for 20 min. The protoplast solution (200 μL of 2 × 10^7^ protoplasts mL^−1^) was then mixed with the assembled RNPs (50 μL) and 5 μL donor template (300–400 ng) and incubated at room temperature for 20 min. Transformation was initiated by addition of 1.6 mL 50% w/v polyethylene glycol (PEG) solution followed by incubation at room temperature for 10 min. The reaction was then halted by addition of 3.2 mL STC buffer. Fungal protoplasts were regenerated by pipetting 475 μL of transformed protoplasts into sterile Petri dishes, covering with approximately 20 mL of regeneration medium (RM) consisting of 239.4 g L^−1^ sucrose, 0.5 g L^−1^ yeast extract and 20 g L^−1^ Bacto agar (BD Difco) and swirling plates to mix. Protoplasts were allowed to regenerate overnight before adding a selection layer of selective RM containing 350 μg mL^−1^ hygromycin on top of the protoplast plates. Plates were then incubated at 25°C with putative mutant Fola4 colonies becoming visible after 3–5 days. Individual mutants were then transferred onto Czapek Dox agar (CDA; Thermo Scientific) amended with 100 μg mL^−1^ hygromycin to confirm phenotype.

Edits at the *SIX8* locus were verified by PCR analysis and Sanger sequencing (Figure S4c, Table S5). Additionally, GFP imaging was used to confirm the fluorescence phenotype of successful mutants (Figure S5).

#### 4.4.2 *Agrobacterium‐*Mediated Complementation of *SIX8* and Insertion of GFP Into Wild‐Type Fola4

*Fusarium oxysporum Agrobacterium* transformation was performed using a protocol based on methods described in Michielse et al. (2009) and Takken et al. (2004). An overnight culture of *A. tumefaciens* EHA105 containing the *SIX8* complementation plasmid cloned as described previously (Figure S4b) was grown in 5 mL of yeast tryptone medium (YT; 16 g L^−1^ tryptone, 5 g L^−1^ yeast extract, 5 g L^−1^ NaCl) supplemented with kanamycin (50 μg mL^−1^) and rifampicin (20 μg mL^−1^). The culture was diluted to a final OD_660_ of 0.45 in induction medium (IM; 0.01 M K_2_HPO_4_, 0.01 M KH_2_PO_4_, 2.5 mM NaCl, 4 mM (NH_4_)_2_SO_4_, 0.5% glycerol (w/v), 2 mM MgSO_4_, 0.7 mM CaCl_2_, 0.009 mM FeSO_4_, 0.01 M glucose, 0.04 M MES, pH 5.3) and the cells incubated for 3 h at 28°C in the presence of acetosyringone (AS; 0.2 mM) prior to use to facilitate fungal transformation.

The Fola4 *SIX8* knockout mutant ΔSIX8 2–14 was selected for complementation. The isolate was cultured in a 250 mL conical flask containing 100 mL of NO_3_ medium (1.7 g L^−1^ yeast nitrogen base [without amino acids; BD Difco], 30 g L^−1^ sucrose, 10.11 g L^−1^ KNO_3_) at 25°C for 5 days. The resulting conidia were harvested by filtration over Miracloth and diluted in IM to a concentration of 2 × 10^6^ spores mL^−1^. Equal volumes of *A. tumefaciens* EHA105 containing the *SIX8* complementation plasmid and ΔSIX8 2–14 spore suspension were mixed and 25 μL spread onto ME‐25 filters (0.45 μm pore size, 47 mm diameter; Schleicher & Schuell) placed on co‐cultivation medium (CM; as IM but with 5 mM glucose, 15 g L^−1^ Bacto agar [BD Difco], 0.2 mM AS) plates. Plates were incubated at 25°C for 2 days and then the filter papers transferred onto CDA plates amended with 0.1 M Tris pH 8, 200 μg mL^−1^ Zeocin (Gibco, Thermo Scientific) and 95.5 μg mL^−1^ cefotaxime for selection of mutants and removal of the *Agrobacterium* culture. Selection plates were then incubated at 25°C for 4–8 days and monospores made for further investigation.

Complementation of *SIX8* was verified by PCR analysis and Sanger sequencing (Figure S4d, Table S5).

### 4.5 Growth Assessments of Fola4 *SIX8* Knockout and Complementation Mutants

To assess if any of the Fola4 AJ516 mutants had reduced fitness due to potential deleterious effects of transformation, the growth of seven *SIX8* knockout and seven complementation mutants was determined on PDA. A core borer was used to excise mycelial plugs (5 mm in diameter) taken from actively growing colonies and placed onto the centre of both PDA plates and PDA plates amended with the selection antibiotics hygromycin (100 μg mL^−1^) or zeocin (200 μg mL^−1^) for *SIX8* knockout or complementation mutants respectively and incubated at 25°C. There were four replicate PDA/PDA + antibiotic plates for each mutant isolate. Mycelial growth was measured along two perpendicular lines drawn from the centre of each mycelial plug to the edge of the plates after 5 days. The wild‐type Fola4 isolate AJ516 was used as a control for comparison with the *SIX8* knockout mutants while both wild‐type AJ516 and the ΔSIX8 2‐14 knockout mutant were used as controls for comparison with the complementation mutants. No significant difference in growth was observed in the knockout mutants compared to wild‐type AJ516 when grown on standard PDA (Figure S2).

### 4.6 Virulence Tests of *SIX8* Mutants on Lettuce Plants

#### 4.6.1 Inoculation of Lettuce Seedlings in an In Vitro System

Virulence of *SIX8* mutants was assessed using the in vitro inoculation bioassay described above; however, about five seeds of cv. Steamboat were used per plate to allow for easier scoring (there was slight variation in seedling germination from plate to plate but no less than three seedlings were present per plate).

Seven Fola4 isolate AJ516 *SIX8* knockout mutants were tested for virulence using the susceptible lettuce cv. Steamboat. Wild‐type Fola4 isolate AJ516, wild‐type Fola1 isolate AJ520 and an uninoculated control comprising 0.012% Tween 20 in SDW were also included. Spore suspensions of each of the treatments were used for inoculation, with 1.5 mL at a concentration of 10^6^ spores mL^−1^ pipetted directly over the lettuce seedling roots in each plate as described above. There were eight replicate plates for each treatment that were left in an incubator at 25°C (16 h photoperiod). Here, disease development was scored over a period of 28 days using a root browning score based on the percentage of total roots affected (Figure S1d).

Additionally, seven Fola4 isolate AJ516 *SIX8* complementation mutants derived from the *SIX8* knockout mutant ΔSIX8 2‐14 were tested for virulence using the susceptible lettuce cv. Steamboat using a similar approach to that outlined above. Wild‐type Fola4 isolate AJ516, ΔSIX8 2‐14 and an uninoculated control comprising 0.012% Tween 20 in SDW were used as controls. Replication of treatments was designed as above and again disease development was scored over a period of 28 days using a root browning score based on the percentage of total roots affected (Figure S1d).

#### 4.6.2 Inoculation of Lettuce in the Glasshouse

Fola4 AJ516 *SIX8* knockout (4), and *SIX8* complementation (4) mutants were assessed for virulence on the susceptible lettuce cv. Steamboat in the glasshouse and compared with the wild‐type Fola4 isolate AJ516 and Fola1 isolate AJ520. Lettuce seed was sown and plants raised in P84 modules for 2–3 weeks using F2S compost (Levington).

Inoculum of each Fola4 isolate was grown in flasks containing a sterile compost‐bran mix, prepared by mixing sieved (4 mm^2^) Levington M2 compost and milled bran (Charlecote Mill, UK) in a ratio of 148.43 g dry compost to 100 g bran and adjusting moisture content to 79%. The compost‐bran mix (~300 g) was transferred into 1 L conical flasks that were then sealed with cotton wool bungs and two layers of aluminium foil, and autoclaved three times at 121°C for 15 min. Flasks were inoculated with five 5 mm^2^ agar plugs excised from the growing edge of actively growing cultures of the different Fola isolate treatments and incubated at 25°C for 4–6 weeks. Inoculum concentrations of each flask were then determined through serial dilution of 1 g inoculum of each treatment. Inoculum of each Fola isolate was then used to amend compost (John Innes No. 3) to achieve a concentration of 1 × 10^6^ CFU g^−1^ for each treatment.

Two‐week‐old lettuce seedlings (cv. Steamboat) were transferred into pots filled with compost‐inoculum mix (10^6^ CFU g^−1^), such that there were 14 replicate pots per Fola isolate treatment that were arranged in a glasshouse compartment in a randomised block design. Plants were maintained at 25°C day/16°C night with a 16 h photoperiod for up to 6 weeks. Plants were assessed for Fusarium wilt symptoms using a scoring system based on percentage leaves wilting (Figure S3c). At harvest at 41 dpi, the main tap root of each harvested plant was cut vertically, and a vascular browning score was recorded based on symptom severity (Figure S3d).

### 4.7 Statistical Analyses

Statistical analyses were performed and figures generated in R Studio (Release ldb809b8, 2022‐05‐16). Differences in disease severity score frequencies between treatment groups were analysed for statistical significance using Kruskal–Wallis, followed by post hoc analysis using Dunn's test with Benjamini–Hochberg adjustment at the 5% significance level. For RT‐qPCR log gene expression and dry weight data differences between treatment groups were analysed for statistical significance using ANOVA followed by post hoc analysis using either Least Significant Difference (LSD; 5% level) or Tukey's HSD, respectively.

## Author Contributions

**Andrew D. Legg:** writing – original draft, writing – review and editing, data curation, investigation, validation, formal analysis, conceptualization, methodology, visualization. **Petra Houterman:** methodology, visualization, resources, writing – review and editing. **Babette V. Vlieger:** methodology, resources, writing – review and editing, visualization. **Martijn Rep:** supervision, funding acquisition, visualization, resources, writing – review and editing. **John P. Clarkson:** supervision, funding acquisition, visualization, project administration, resources, writing – review and editing, writing – original draft, conceptualization.

## Funding

This work was supported by the Biotechnology and Biological Sciences Research Council. Agriculture and Horticulture Development Board.

## Conflicts of Interest

The authors declare no conflicts of interest.

## Supporting information


**Figure S1:** In vitro lettuce seedling bioassays. Mean root browning disease score for seven Fola4 AJ516 *SIX8* knockout mutants (a), seven Fola4 *SIX8* complementation mutants (b), compared with wild‐type (WT) Fola4 isolate AJ516 and Fola1 isolate AJ520 over 28 days. Error bars indicate the least significant difference (LSD) at 5% level. (c) Root browning symptoms following inoculation with WT Fola4 AJ516, a Fola4 *SIX8* complementation mutant (SIX8comp32), a *SIX8* knockout mutant (ΔSIX8 2‐14) and an uninoculated control. (d) Fusarium disease scoring system for Fola‐inoculated lettuce seedlings based on percentage root area with browning: 0, healthy seedling; 1, 1%–25%; 2, 26%–50%; 3, 51%–75%; 4, > 75%; 5, plant death.

**Figure S2:** Mean colony radius after 5 days at 25°C for *Fusarium oxysporum* f. sp. *lactucae* race 4 (Fola4) isolate AJ516 *SIX8* knockout (a, b) complementation (c, d) mutants and wild‐type (WT) Fola4 on (a) potato dextrose agar (PDA), (b) PDA + hygromycin (100 μg mL^−1^), (c) PDA, (d) PDA + zeocin (200 μg mL^−1^). Level of significance following Tukey HSD comparisons of treatments with WT Fola4 AJ516; ns = not significant, ****p < 0.0001.

**Figure S3:** Glasshouse lettuce plant bioassays. (a) Mean wilt disease score for four Fola4 *SIX8* knockout mutants, four *SIX8* complementation mutants, wild‐type Fola4 isolate AJ516 and Fola1 isolate AJ520 over 38 days. Error bars indicate the least significant difference (LSD) at 5% level; (b) Mean dry weight of lettuce heads at 42 days post‐treatment. Groups denoted by the same letter indicate no significant difference as determined by ANOVA followed by Tukey's HSD post hoc analysis at the 5% significance level. (c, d) *Fusarium* disease scoring system for Fola‐inoculated pot‐grown glasshouse lettuce based on percentage lettuce leaf wilt: 0, healthy plant; 1, wilting of 1–2 leaves; 2, 10%–50%; 3, > 50%; 4, 100%; 5, plant death (c); and (d) vascular browning at harvest based on: 0, no symptoms; 1, mild vascular browning; 2, vascular browning; 3, severe vascular browning; 4, plant death (not shown).

**Figure S4:** Infographics for *SIX8* knockout and complementation constructs. (a) CRISPR *SIX8* knockout plasmid (pink lines and labels indicate primer names and annealing sites used for amplification of linear donor DNA in the transformation protocol); (b) the final assembled *SIX8* complementation plasmid; (c) the CRISPR‐Cas9 transformed *SIX8* knockout locus. Four pairs of primers (A–D, Table S5) were used to amplify regions to confirm successful insertion of the donor DNA into *SIX8* knockout mutants. (A) Left genomic region into selection cassette (primers 10,322/751), (B) selection cassette into right genomic region (primers 745/SIX8 flanks rev), (C) absence of the wild type *SIX8* gene and the presence of full donor DNA insert (primers 17,722/SIX8 flanks rev), (D) presence of the hygromycin phosphotransferase (hph) gene (primers 1605/8251); (d) *Agrobacterium*‐mediated ectopic transformation of the *SIX8* complementation T‐DNA locus. Three pairs of primers (A–C, Table S5) were used to amplify regions to confirm successful insertion of T‐DNA into putative *SIX8* complementation mutants. (A) presence of *SIX8* (primers Fola4 SIX8 F1/Fola4 SIX8 R1); (B) presence of phleomycin resistance gene (ble) (primers 3657/87); (C) presence of the *SIX8* promoter (primers used Left flank fwd (SIX8comp)/Fola4 SIX8 R1).

**Figure S5:** Bright field and GFP fluorescent images of two putative *Fusarium oxysporum* f. sp. *lactucae* race 4 (Fola4) *SIX8* knockout mutants compared to the AJ516 wild‐type isolate.

**Table S1:** Expression of *Secreted In Xylem* (*SIX*) genes over time as determined by reverse transcription‐quantitative PCR in roots of lettuce cultivar Steamboat following infection with *Fusarium oxysporum* f. sp. *lactucae* race 4 (Fola4) isolate AJ516. Expression values were calculated relative to *Translation Elongation Factor 1a* (*TEF*) at eight time points (0–120 h post‐inoculation). Values represent log‐transformed means from ANOVA.

**Table S2:** In vitro lettuce seedling bioassays statistics. Kruskal–Wallis tests followed by Dunn's post hoc analysis with Benjamini–Hochberg adjustment at the 5% significance level for pairwise comparisons of root browning disease scores following inoculation of lettuce plants with Fola4 wild type, *SIX8* knockout mutants (a), *SIX8* complementation mutants (b), as well as a Fola1 isolate. Values represent adjusted *p* values for differences between treatments. Significance levels are indicated using asterisks (*p*_adj_.signif), with thresholds as follows: ns = not significant (*p* ≥ 0.05), *p < 0.05, **p < 0.01, ***p < 0.001, ****p < 0.0001.

**Table S3:** Glasshouse lettuce plant bioassays statistics. Kruskal–Wallis tests followed by Dunn's post hoc analysis with Benjamini–Hochberg adjustment at the 5% significance level for pairwise comparisons of leaf wilt disease scores (a) and vascular browning disease scores (b) following inoculation of lettuce plants with Fola4 wild type, *SIX8* knockout and *SIX8* complementation mutants as well as a Fola1 isolate. ANOVA followed by Tukey's HSD post hoc analysis at the 5% significance level for pairwise comparisons of lettuce head mean dry weights (c). Values represent adjusted *p* values for differences between treatments. Significance levels are indicated using asterisks (*p*_adj_.signif), with thresholds as follows: ns = not significant (*p* ≥ 0.05), *p < 0.05, **p < 0.01, ***p < 0.001, ****p < 0.0001.

**Table S4:** Primers used for reverse transcription‐quantitative PCR of *SIX* genes and *TEF* in *Fusarium oxysporum* f. sp. *lactucae* isolate AJ516.

**Table S5:** Oligomers and primer pairs used in the production of *SIX8* sgRNAs, the assembly of the *SIX8* knockout and complementation donor DNA plasmids and for confirmation of the *Fusarium oxysporum* f. sp. *lactucae* race 4 isolate AJ516 *SIX8* knockout and complementation mutants.

**Table S6:** Sequences of two designed *SIX8* specific sgRNAs used for CRISPR‐Cas9 knockout of *SIX8* in the *Fusarium oxysporum* f. sp. *lactucae* race 4 isolate AJ516.

## Data Availability

The data that support the findings of this study are available from the corresponding author upon reasonable request.
