# Supplementary material for: The Effector SIX8 Contributes to Virulence of Fusarium oxysporum f. sp. lactucae Race 4 on Lettuce
Source: Mol Plant Pathol. 2026 Jun 9;27(6):e70296. doi: 10.1111/mpp.70296 (PMC13250395; doi:10.1111/mpp.70296)
Supplement: Supplementary file 1 — Figure S1: In vitro lettuce seedling bioassays. Mean root browning disease score for seven Fola4 AJ516 SIX8 knockout mutants (a), seven Fola4 SIX8 complementation mutants (b), compared with wild‐type (WT) Fola4 isolate AJ516 and Fola1 isolate AJ520 over 28 days. Error bars indicate the least significant difference (LSD) at 5% level. (c) Root browning symptoms following inoculation with WT Fola4 AJ516, a Fola4 SIX8 complementation mutant (SIX8comp32), a SIX8 knockout mutant (ΔSIX8 2‐14) and an uninoculated control. (d) Fusarium disease scoring system for Fola‐inoculated lettuce seedlings based on percentage root area with browning: 0, healthy seedling; 1, 1%–25%; 2, 26%–50%; 3, 51%–75%; 4, > 75%; 5, plant death. [file MPP-27-e70296-s003.pdf]

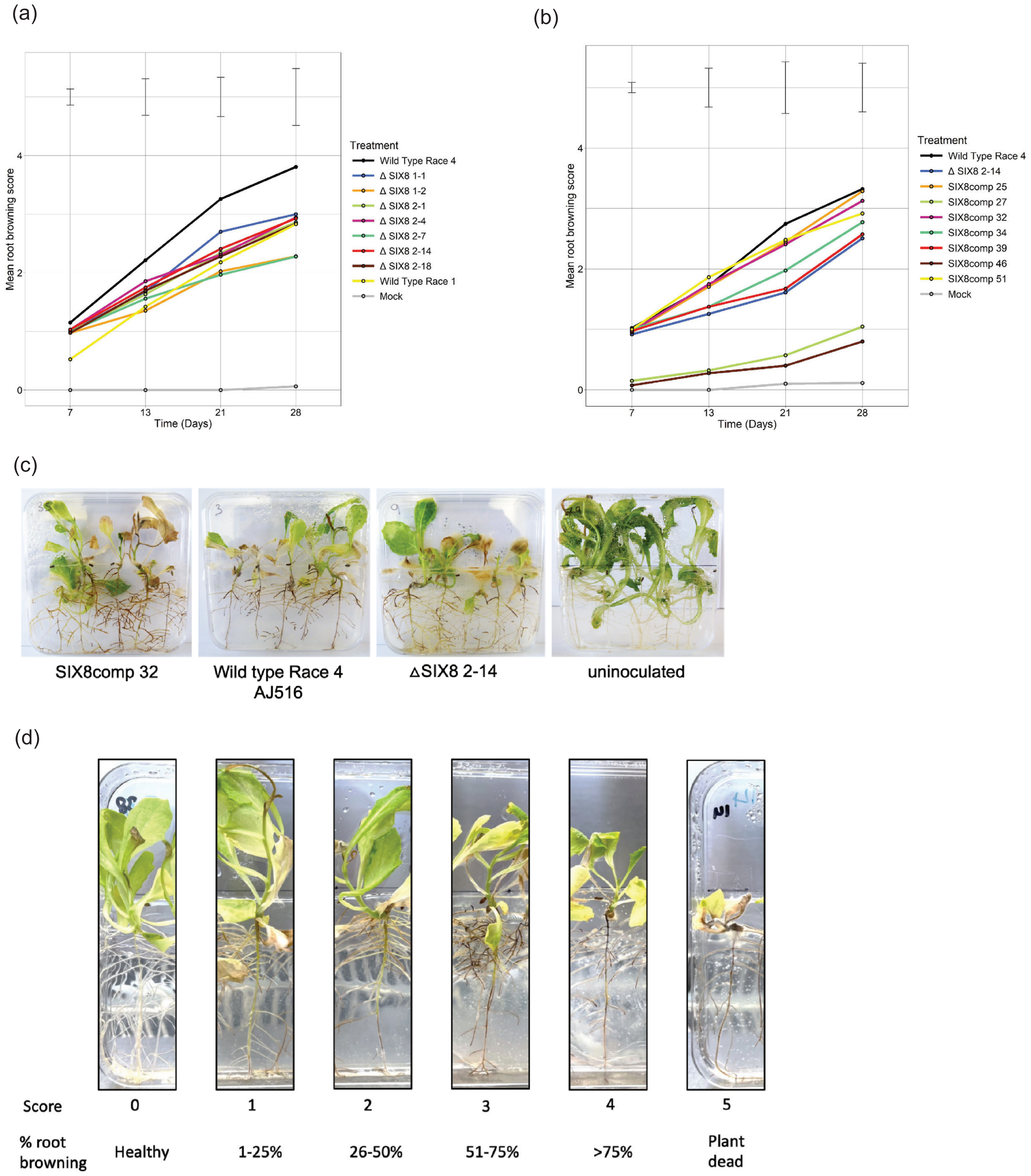

Figure S1 *In vitro* lettuce seedling bioassays. Mean root browning disease score for seven Fola4 AJ516 *SIX8* knockout mutants (a), seven Fola4 *SIX8* complementation mutants (b), compared with wild type (WT) Fola4 isolate AJ516 and Fola1 isolate AJ520 over 28 days. Error bars indicate the least significant difference (LSD) at 5% level. (c) Root browning symptoms following inoculation with WT Fola4 AJ516, a Fola4 *SIX8* complementation mutant (SIX8comp32), a *SIX8* knockout mutant (ΔSIX8 2-14), and an uninoculated control. (d) *Fusarium* disease scoring system for Fola-inoculated lettuce seedlings based on percentage root area with browning: 0, healthy seedling; 1, 1-25%; 2, 26-50%; 3, 51-75%; 4, >75%; 5, plant death.
