# Supplementary material for: The Effector SIX8 Contributes to Virulence of Fusarium oxysporum f. sp. lactucae Race 4 on Lettuce
Source: Mol Plant Pathol. 2026 Jun 9;27(6):e70296. doi: 10.1111/mpp.70296 (PMC13250395; doi:10.1111/mpp.70296)
Supplement: Supplementary file 2 — Figure S2: Mean colony radius after 5 days at 25°C for Fusarium oxysporum f. sp. lactucae race 4 (Fola4) isolate AJ516 SIX8 knockout (a, b) complementation (c, d) mutants and wild‐type (WT) Fola4 on (a) potato dextrose agar (PDA), (b) PDA + hygromycin (100 μg mL−1), (c) PDA, (d) PDA + zeocin (200 μg mL−1). Level of significance following Tukey HSD comparisons of treatments with WT Fola4 AJ516; ns = not significant, ****p < 0.0001. [file MPP-27-e70296-s002.pdf]

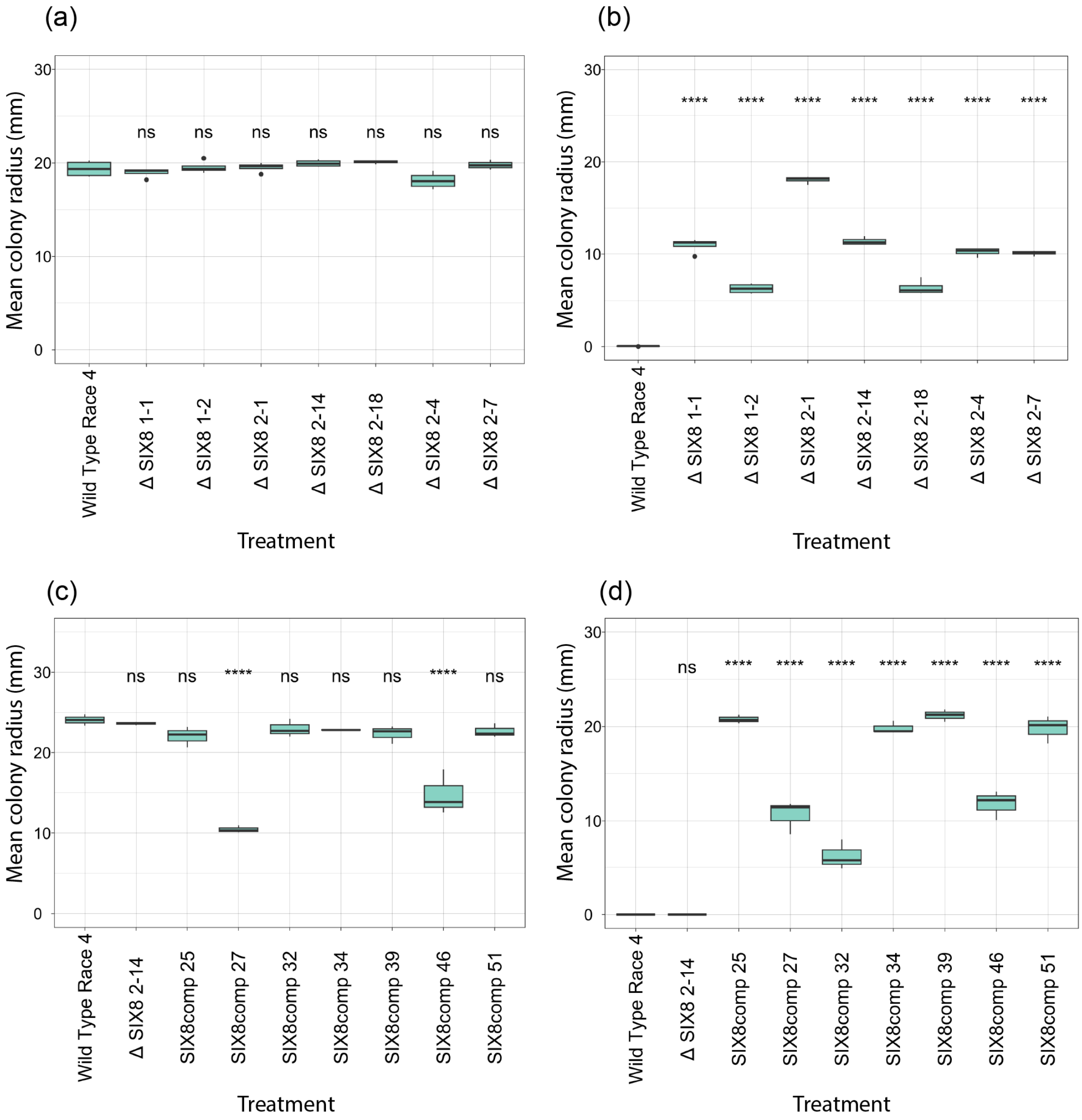

Figure S2 Mean colony radius after 5 days at 25°C for *Fusarium oxysporum* f. sp. *lactucae* race 4 (Fola4) isolate AJ516 *SIX8* knockout (a, b) complementation (c, d) mutants and WT Fola4 on a) PDA, b) PDA + hygromycin (100 μg mL^{-1}), c) PDA, d) PDA + zeocin (200 μg mL^{-1}). Level of significance following Tukey HSD comparisons of treatments with wild type Fola4 AJ516; ns = not significant, **** $p < 0.0001$.
