# Supplementary material for: The Effector SIX8 Contributes to Virulence of Fusarium oxysporum f. sp. lactucae Race 4 on Lettuce
Source: Mol Plant Pathol. 2026 Jun 9;27(6):e70296. doi: 10.1111/mpp.70296 (PMC13250395; doi:10.1111/mpp.70296)
Supplement: Supplementary file 3 — Figure S3: Glasshouse lettuce plant bioassays. (a) Mean wilt disease score for four Fola4 SIX8 knockout mutants, four SIX8 complementation mutants, wild‐type Fola4 isolate AJ516 and Fola1 isolate AJ520 over 38 days. Error bars indicate the least significant difference (LSD) at 5% level; (b) Mean dry weight of lettuce heads at 42 days post‐treatment. Groups denoted by the same letter indicate no significant difference as determined by ANOVA followed by Tukey's HSD post hoc analysis at the 5% significance level. (c, d) Fusarium disease scoring system for Fola‐inoculated pot‐grown glasshouse lettuce based on percentage lettuce leaf wilt: 0, healthy plant; 1, wilting of 1–2 leaves; 2, 10%–50%; 3, > 50%; 4, 100%; 5, plant death (c); and (d) vascular browning at harvest based on: 0, no symptoms; 1, mild vascular browning; 2, vascular browning; 3, severe vascular browning; 4, plant death (not shown). [file MPP-27-e70296-s004.pdf]

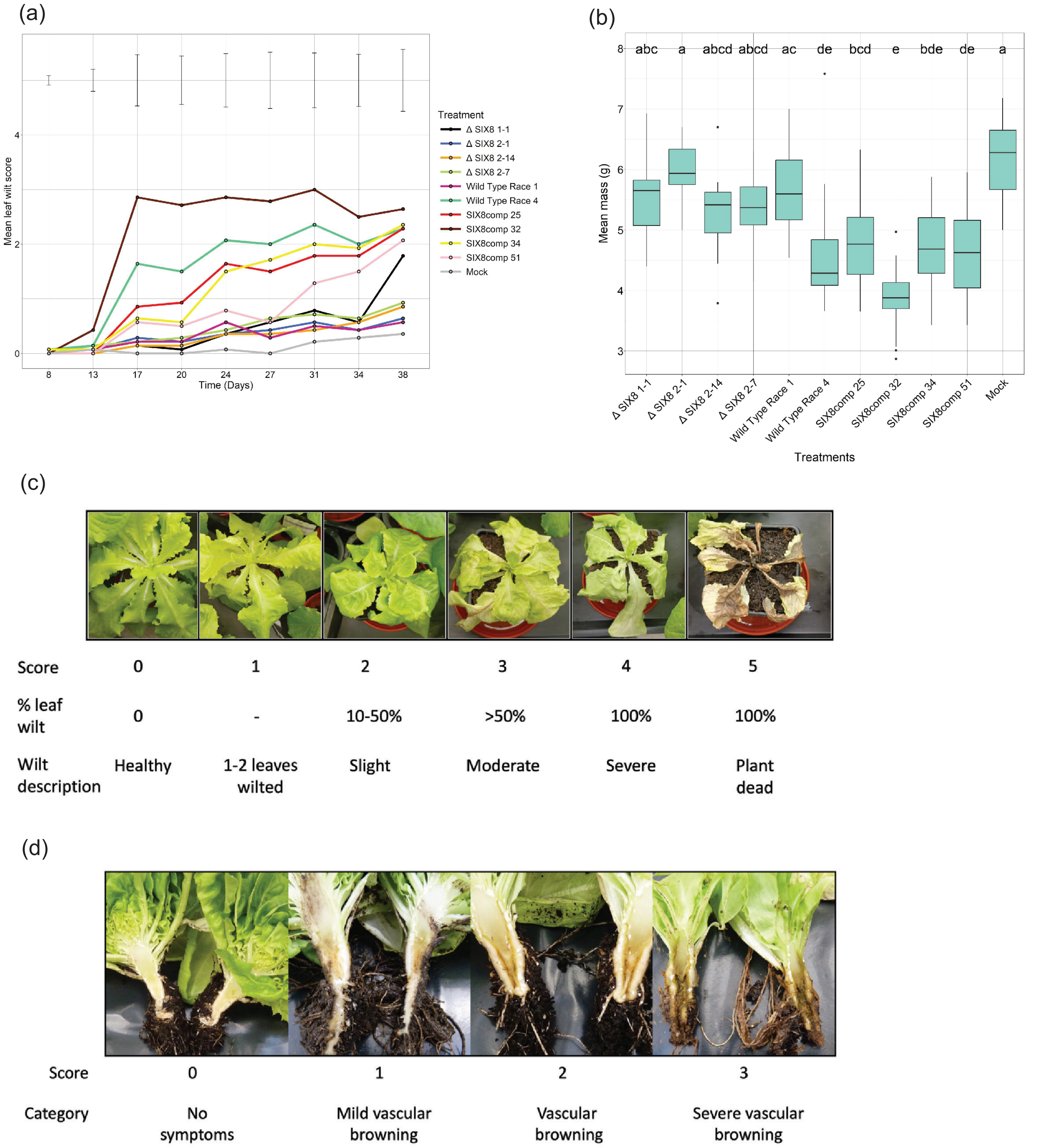

Figure S3 Glasshouse lettuce plant bioassays. (a) Mean wilt disease score for four Fola4 *SIX8* knockout mutants, four *SIX8* complementation mutants, wild type Fola4 isolate AJ516 and Fola1 isolate AJ520 over 38 days. Error bars indicate the least significant difference (LSD) at 5% level; (b) Mean dry weight of lettuce heads at 42 dpi post treatment. Groups denoted by the same letter indicate no significant difference as determined by the Analysis of Variance test followed by Tukey's HSD post hoc analysis at the 5% significance level. (c and d) *Fusarium* disease scoring system for Fola-inoculated pot-grown glasshouse lettuce based on percentage lettuce leaf wilt: 0, healthy plant; 1, wilting of 1-2 leaves; 2, 10-50%; 3, >50%; 4, 100%; 5, plant death (c); and (d) vascular browning at harvest based on: 0, no symptoms; 1, mild vascular browning; 2, vascular browning; 3, severe vascular browning; 4, plant death (not shown).
