# Supplementary material for: The Effector SIX8 Contributes to Virulence of Fusarium oxysporum f. sp. lactucae Race 4 on Lettuce
Source: Mol Plant Pathol. 2026 Jun 9;27(6):e70296. doi: 10.1111/mpp.70296 (PMC13250395; doi:10.1111/mpp.70296)
Supplement: Supplementary file 4 — Figure S4: Infographics for SIX8 knockout and complementation constructs. (a) CRISPR SIX8 knockout plasmid (pink lines and labels indicate primer names and annealing sites used for amplification of linear donor DNA in the transformation protocol); (b) the final assembled SIX8 complementation plasmid; (c) the CRISPR‐Cas9 transformed SIX8 knockout locus. Four pairs of primers (A–D, Table S5) were used to amplify regions to confirm successful insertion of the donor DNA into SIX8 knockout mutants. (A) Left genomic region into selection cassette (primers 10,322/751), (B) selection cassette into right genomic region (primers 745/SIX8 flanks rev), (C) absence of the wild type SIX8 gene and the presence of full donor DNA insert (primers 17,722/SIX8 flanks rev), (D) presence of the hygromycin phosphotransferase (hph) gene (primers 1605/8251); (d) Agrobacterium‐mediated ectopic transformation of the SIX8 complementation T‐DNA locus. Three pairs of primers (A–C, Table S5) were used to amplify regions to confirm successful insertion of T‐DNA into putative SIX8 complementation mutants. (A) presence of SIX8 (primers Fola4 SIX8 F1/Fola4 SIX8 R1); (B) presence of phleomycin resistance gene (ble) (primers 3657/87); (C) presence of the SIX8 promoter (primers used Left flank fwd (SIX8comp)/Fola4 SIX8 R1). [file MPP-27-e70296-s008.pdf]

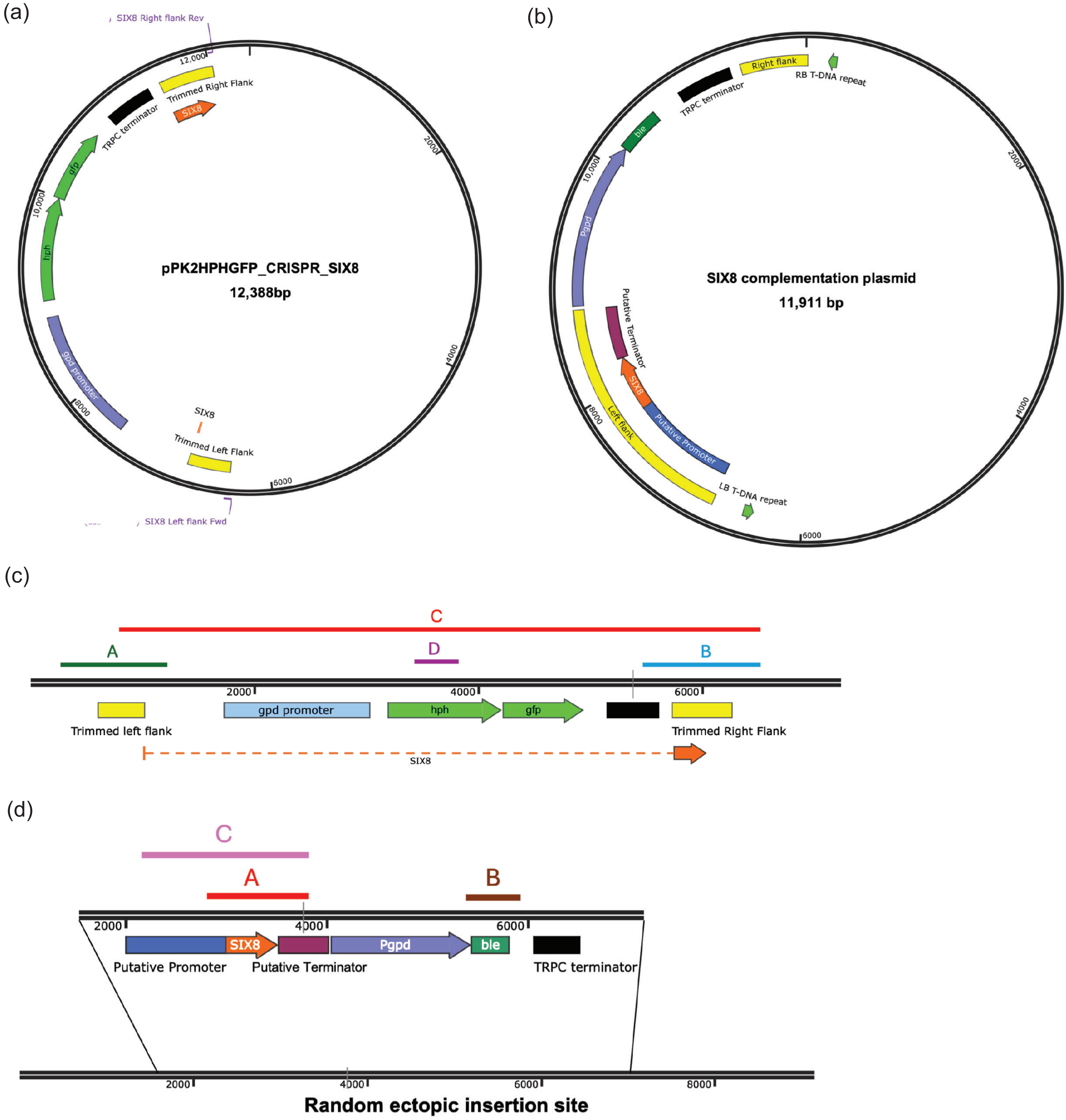

Figure S4 Infographics for *SIX8* knockout and complementation constructs. (a) CRISPR *SIX8* knockout plasmid (pink lines and labels indicate primer names and annealing sites used for amplification of linear donor DNA in the transformation protocol); (b) the final assembled *SIX8* complementation plasmid; (c) the CRISPR-Cas9 transformed *SIX8* knockout locus. Four pairs of primers (A-D, Table S5) were used to amplify regions to confirm successful insertion of the donor DNA into *SIX8* knockout mutants. A) left genomic region into selection cassette (primers 10322/751), B) selection cassette into right genomic region (primers 745/SIX8 flanks rev), C) absence of the wild type *SIX8* gene and the presence of full donor DNA insert (primers 17722/SIX8 flanks rev), D) presence of the hygromycin phosphotransferase (hph) gene (primers 1605/8251); (d) *Agrobacterium*-mediated ectopic transformation of the *SIX8* complementation T-DNA locus. Three pairs of primers (A-C, Table S5) were used to amplify regions to confirm successful insertion of T-DNA into putative *SIX8* complementation mutants. A) presence of *SIX8* (primers Fola4 SIX8 F1 /Fola4 SIX8 R1); B) presence of phleomycin resistance gene (ble) (primers 3657/87); C) presence of the *SIX8* promoter (primers used Left flank fwd (SIX8comp)/ Fola4 SIX8 R1).
