# Supplementary material for: The Effector SIX8 Contributes to Virulence of Fusarium oxysporum f. sp. lactucae Race 4 on Lettuce
Source: Mol Plant Pathol. 2026 Jun 9;27(6):e70296. doi: 10.1111/mpp.70296 (PMC13250395; doi:10.1111/mpp.70296)
Supplement: Supplementary file 5 — Figure S5: Bright field and GFP fluorescent images of two putative Fusarium oxysporum f. sp. lactucae race 4 (Fola4) SIX8 knockout mutants compared to the AJ516 wild‐type isolate. [file MPP-27-e70296-s001.pdf]

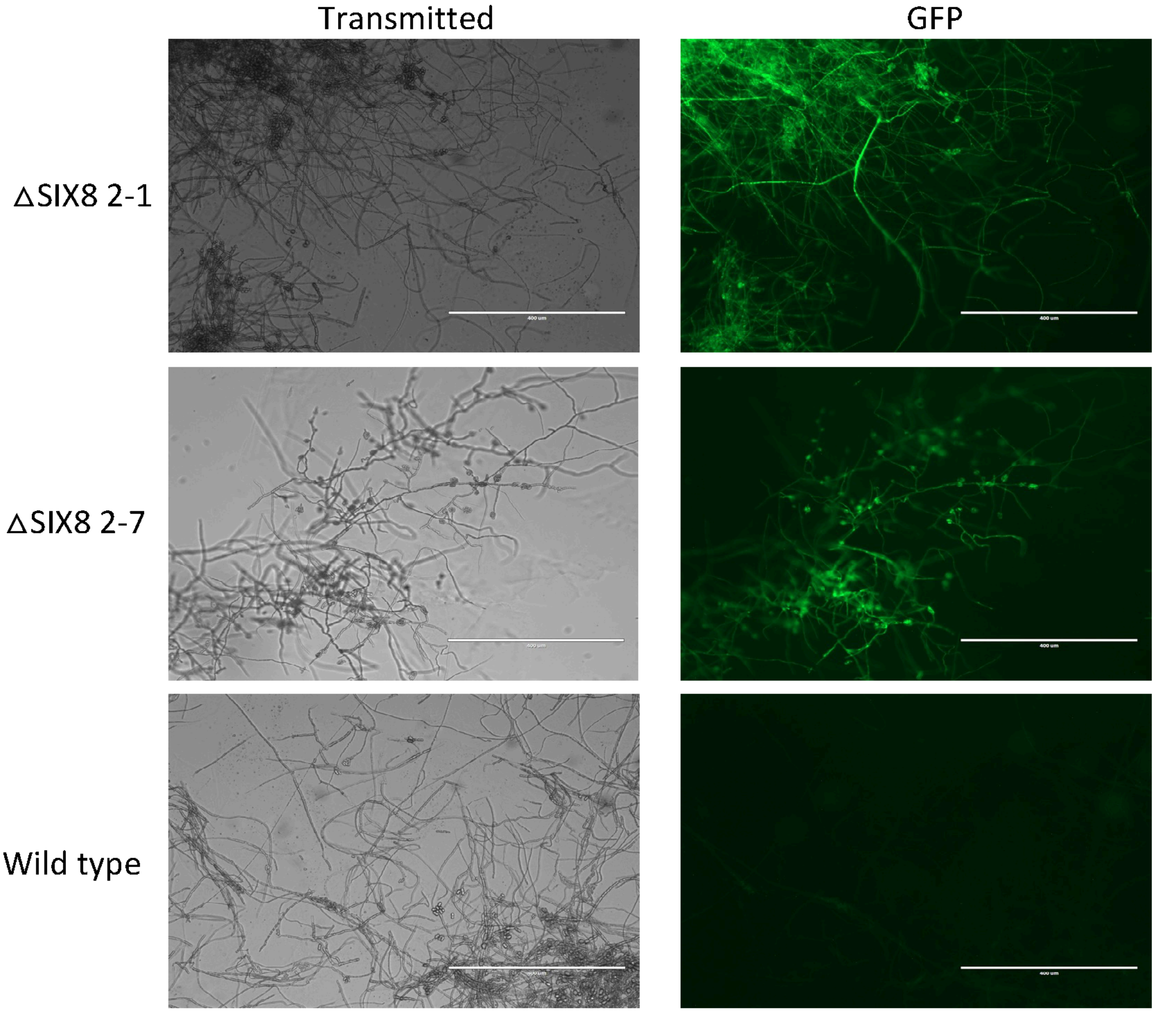


**Figure S5** Bright field and GFP fluorescent images of two *Fusarium oxysporum* f. sp. *lactucae* race 4 (Fola4) *SIX8* knockout mutants compared to the AJ516 wild type isolate.
