# Supplementary material for: The Effector SIX8 Contributes to Virulence of Fusarium oxysporum f. sp. lactucae Race 4 on Lettuce
Source: Mol Plant Pathol. 2026 Jun 9;27(6):e70296. doi: 10.1111/mpp.70296 (PMC13250395; doi:10.1111/mpp.70296)
Supplement: Supplementary file 6 — Table S1: Expression of Secreted In Xylem (SIX) genes over time as determined by reverse transcription‐quantitative PCR in roots of lettuce cultivar Steamboat following infection with Fusarium oxysporum f. sp. lactucae race 4 (Fola4) isolate AJ516. Expression values were calculated relative to Translation Elongation Factor 1a (TEF) at eight time points (0–120 h post‐inoculation). Values represent log‐transformed means from ANOVA. [file MPP-27-e70296-s009.pdf]

**Table S1** Expression of *Secreted In Xylem* (*SIX*) genes over time as determined by qPCR in roots of lettuce cultivar Steamboat following infection with *Fusarium oxysporum* f. sp. *lactucae* race 4 (Fola4) isolate AJ516. Expression values were calculated relative to *Translation Elongation Factor 1a* (*TEF*) at eight time points (0 - 120 hours post inoculation). Values represent log transformed means from ANOVA analysis.

| Time (h) | Relative expression of *SIX* gene to *TEF* (log transformed means) | | | |
|---|---|---|---|---|
| | *SIX8* | *SIX9* | *SIX14* | *PSL1* |
| **0** | *NDt* | *NDt* | *NDt* | *NDt* |
| **6** | *NDt* | *NDt* | *NDt* | *NDt* |
| **12** | *NDt* | -2.04 | -3.43 | *NDt* |
| **24** | -1.95 | -0.66 | -1.53 | *NDt* |
| **48** | -0.52 | -0.21 | -0.99 | -2.61 |
| **72** | 0.06 | 0.33 | -0.41 | -1.89 |
| **96** | 0.23 | 0.61 | -0.38 | -1.55 |
| **120** | 0.47 | 0.81 | -0.34 | -1.19 |
| **LSD[a]** | 0.70 | 0.43 | 0.17 | 0.80 |
| **LSD[b]** | 0.28 | | | |

[a] LSD (5% level) used to calculate significant differences between time points. *NDt* (not detected) denotes undetectable levels of expression by qPCR. [b] LSD (5% level) used to calculate significant differences between *SIX* and *PSL1* genes at 120 hours post infection.
