# Supplementary material for: The Effector SIX8 Contributes to Virulence of Fusarium oxysporum f. sp. lactucae Race 4 on Lettuce
Source: Mol Plant Pathol. 2026 Jun 9;27(6):e70296. doi: 10.1111/mpp.70296 (PMC13250395; doi:10.1111/mpp.70296)
Supplement: Supplementary file 9 — Table S4: Primers used for reverse transcription‐quantitative PCR of SIX genes and TEF in Fusarium oxysporum f. sp. lactucae isolate AJ516. [file MPP-27-e70296-s006.pdf]

**Table S4** Primers used for RT-qPCR of *SIX* genes and *TEF* in *Fusarium oxysporum* f. sp. *lactucae* isolate AJ516.

| Gene | Primer pairs | Sequence 5'-3' (forward/reverse) | Annealing temp. (°C) |
|---|---|---|---|
| *TEF*[a] | qTEF F2/qTEF R2 | GGTCAGGTCGGTGCTGGTTACG/TGGATCTCGGCGAACTTGCAGG | 63 |
| *SIX8*[b] | Fola4 *SIX8* F1/Fola4 *SIX8* R1 | ACGTTGAGGGTGGACAGAAC/TCGTGTACCGCTTGTGAGAG | 59 |
| *SIX9*[b] | Fola *SIX9* F2/Fola *SIX9* R2 | CTAGCCCAAGGAGTTGCGGT/GCATTGTCCCATACTGAATCC | 59 |
| *SIX14*[c] | Fola*SIX14*_Exon1F/Fola*SIX14*_Exon1R | GCATTTCCACTATGTATTTCTTC/AACCACCACCTGCGTCTAG | 58 |

[a] Taylor et al., (2016); [b] This study; [c] Dr. Helen Bates (unpublished).

Taylor, A., Vagany, V., Jackson, A. C., Harrison, R. J., Rainoni, A. and Clarkson, J. P. (2016) Identification of pathogenicity-related genes in Fusarium oxysporum f. sp. cepae. Molecular Plant Pathology, 17, 1032-1047.
