# Supplementary material for: The Effector SIX8 Contributes to Virulence of Fusarium oxysporum f. sp. lactucae Race 4 on Lettuce
Source: Mol Plant Pathol. 2026 Jun 9;27(6):e70296. doi: 10.1111/mpp.70296 (PMC13250395; doi:10.1111/mpp.70296)
Supplement: Supplementary file 10 — Table S5: Oligomers and primer pairs used in the production of SIX8 sgRNAs, the assembly of the SIX8 knockout and complementation donor DNA plasmids and for confirmation of the Fusarium oxysporum f. sp. lactucae race 4 isolate AJ516 SIX8 knockout and complementation mutants. [file MPP-27-e70296-s005.pdf]

Table S5 Oligomers and primer pairs used in the production of *SIX8* sgRNAs, the assembly of the *SIX8* knockout and complementation donor DNA plasmids, and for confirmation of the *Fusarium oxysporum* f. sp. *lactucae* race 4 isolate AJ516 *SIX8* knockout and complementation mutants.

Primer pairs \ Oligomers	Sequence 5'-3' (forward/reverse)	Annealing temp. (°C)
**Oligomers for sgRNA synthesis**		
SIX8 sgRNA1 oligo[a]	TTCTAATACGACTCACTATAGCAGCCACAGAGACGGCTAAGTTTTAGAGCTAGA	37
SIX8 sgRNA2 oligo[a]	TTCTAATACGACTCACTATAGGAAGAGTAAAAGAACGCGTGTTTTAGAGCTAGA	37
sgRNA universal oligo[a]	AAAAGCACCGACTCGGTGCCACTTTTTCAAGTTGATAACGGACTAGCCTTATTTTAACTTGCTATTTCTAGCTCTAAAAC	37
**Primers for HiFi assembly of *SIX8* knockout donor DNA plasmid**		
SIX8 Left flank Fwd[a] / *SIX8* Left flank Rev[a]	ATGATTACGAATTCTTAATTAAGATTTATGTAATATTAAGACCGAGAAGG / AAGATCCCCGGGTACCGAGCTCGATCGTAGGGGTTGCATAGCC	62.8
SIX8 Right flank Fwd[a] / *SIX8* Right flank Rev[a]	TCTCCACTCGACCTGCAGGCATGCAGCCGTCTCTGTGGCTGCTAC / CGTTGTAAAACGACGGCCAGTGCCATTATCCCTATCGGGCCTAATCC	62.8
**Primers for CRISPR genotyping**		
11772[b] / *SIX8* flanks rev[a]	ACATCCTTTCGTACCGCATC / CAGCATCCATATCCACGCCATA	55
1605[b] / 8251[b]	GATGTAGGAGGGCGTGGATA / CGTCTGCTGCTCCATACAAG	57
10322[b] / 751[b]	ATAGTTGGGCAGAACGCAGG / CCTTCAGCGGATGATCGACTG	57
745[b] / *SIX8* flanks rev[a]	GCATGTTTCTTCCTTGAACTCTC / CAGCATCCATATCCACGCCATA	61
**Primers used for HiFi assembly of *Agrobacterium* mediated complementation plasmid**		
Left Flank fwd (SIX8comp)[a] / Left Flank rev (SIX8comp)[a]	ATGATTACGAATTCTTAATTAAGATGGCAACACGAGAAGTTAGG / AGGATCCCCGGGTACCGAGCTCGATTACGAATACTGGCACATAAAGC	59
Right Flank fwd (SIX8comp)[a] / Right Flank rev (SIX8comp)[a]	GACCTGCAGAGGCCTAGGCGCGCCAGGGATCTGTGACAGAAGC / CGTTGTAAAACGACGGCCAGTGCCATGCCGACCTATCTTTCTTC	58
**Primers for *Agrobacterium* mediated complementation genotyping**		
3657[b] / 87[b]	CAAGTTGACCAGTGCCGTTC / GATCTCAAGCTCCTGGGAC	55
Left Flank fwd (SIX8comp)[a] / Fola4 *SIX8* R1[a]	ATGATTACGAATTCTTAATTAAGATGGCAACACGAGAAGTTAGG / TCGTGTACCGCTTGTGAGAG	64
Fola4 *SIX8* F1 (PCR)[a] / Fola4 *SIX8* R1 (PCR)[a]	CGCATCAAGAGTCCGGGTTTAC / CAGCATCCATATCCACGCCATA	59

[a] Primers / oligos designed in this study
[b] Primers / oligos designed by Molecular Plant Pathology Department, University of Amsterdam
