# Supplementary material for: The Effector SIX8 Contributes to Virulence of Fusarium oxysporum f. sp. lactucae Race 4 on Lettuce
Source: Mol Plant Pathol. 2026 Jun 9;27(6):e70296. doi: 10.1111/mpp.70296 (PMC13250395; doi:10.1111/mpp.70296)
Supplement: Supplementary file 11 — Table S6: Sequences of two designed SIX8 specific sgRNAs used for CRISPR‐Cas9 knockout of SIX8 in the Fusarium oxysporum f. sp. lactucae race 4 isolate AJ516. [file MPP-27-e70296-s011.pdf]

Table S6 Sequences of two designed *SIX8* specific sgRNAs utilised for CRISPR-Cas9 knockout of *SIX8* in the *Fusarium oxysporum* f. sp. *lactucae* race 4 isolate AJ516.

**sgRNA**	**Sequence 5'-3'** [a]
SIX8 sgRNA1	GCAGCCACAGAGACGGCUAAGUUUUAGAGCUAGAAAUAGCAAGUUAAAAUAAGGCUAGUCCGUUAUCAACUUGAAAAAGUGGCACCGAGUCGGUGCUUUU
SIX8 sgRNA2	GGAAGAGUAAAAGAACGCGUGUUUUAGAGCUAGAAAUAGCAAGUUAAAAUAAGGCUAGUCCGUUAUCAACUUGAAAAAGUGGCACCGAGUCGGUGCUUUU

[a] Colours: green represents sgRNA target sequences; purple represents sgRNA Cas9 scaffold sequences
